# Fascin actin-bundling protein 1 in human cancer: promising biomarker or therapeutic target?

**DOI:** 10.1016/j.omto.2020.12.014

**Published:** 2021-01-20

**Authors:** Hongliang Liu, Yu Zhang, Li Li, Jimin Cao, Yujia Guo, Yongyan Wu, Wei Gao

**Affiliations:** 1 Shanxi Key Laboratory of Otorhinolaryngology Head and Neck Cancer, First Hospital of Shanxi Medical University, Taiyuan 030001, Shanxi, PR China; 2 Shanxi Province Clinical Medical Research Center for Precision Medicine of Head and Neck Cancer, First Hospital of Shanxi Medical University, Taiyuan 030001, Shanxi, PR China; 3 Department of Otolaryngology Head & Neck Surgery, First Hospital of Shanxi Medical University, Taiyuan 030001, Shanxi, PR China; 4 Key Laboratory of Cellular Physiology, Ministry of Education, Shanxi Medical University, Taiyuan 030001, Shanxi, PR China; 5 Department of Cell Biology and Genetics, School of Basic Medical Sciences, Shanxi Medical University, Taiyuan 030001, Shanxi, PR China; 6 Department of Physiology, Shanxi Medical University, Taiyuan 030001, Shanxi, PR China; 7 Department of Biochemistry & Molecular Biology, Shanxi Medical University, Taiyuan 030001, Shanxi, PR China

**Keywords:** FSCN1, cancer, biomarker, therapeutic target, metastasis

## Abstract

Fascin actin-bundling protein 1 (FSCN1) is a highly conserved actin-bundling protein that cross links F-actin microfilaments into tight, parallel bundles. Elevated FSCN1 levels have been reported in many types of human cancers and have been correlated with aggressive clinical progression, poor prognosis, and survival outcomes. The overexpression of FSCN1 in cancer cells has been associated with tumor growth, migration, invasion, and metastasis. Currently, FSCN1 is recognized as a candidate biomarker for multiple cancer types and as a potential therapeutic target. The aim of this study was to provide a brief overview of the FSCN1 gene and protein structure and elucidate on its actin-bundling activity and physiological functions. The main focus was on the role of FSCN1 and its upregulatory mechanisms and significance in cancer cells. Up-to-date studies on FSCN1 as a novel biomarker and therapeutic target for human cancers are reviewed. It is shown that FSCN1 is an unusual biomarker and a potential therapeutic target for cancer.

## Graphical Abstract

**Figure:**
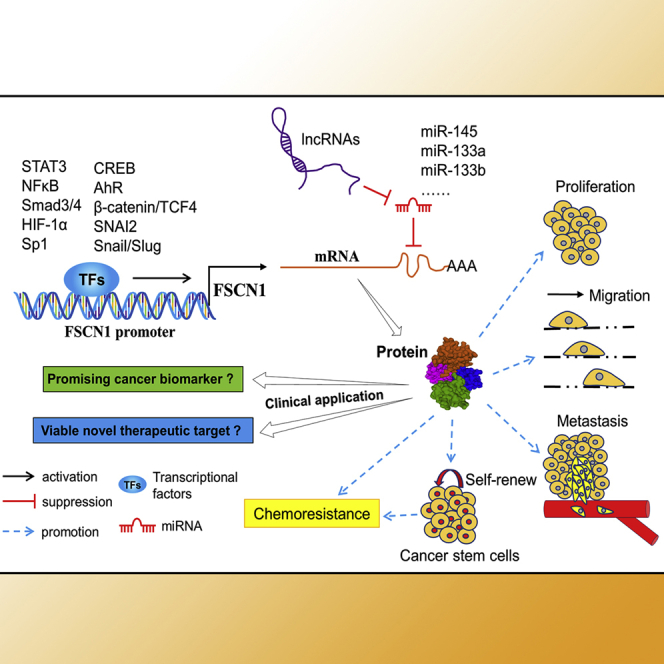

## Teaser

As an actin-bundling protein, FSCN1 plays an important role in cancer progression and metastasis. Its expression has been correlated with an aggressive clinical course and poor prognosis. This review summarizes not only the role of FSCN1 in cancers but also its clinical application in cancer therapy by targeting FSCN1.

## Main text

Fascin actin-bundling protein 1 (FSCN1), also known as fascin1 or fascin, is a globular filamentous actin-binding protein of the fascin family.^1^^,^^2^ By stabilizing actin bundles, FSCN1 supports a variety of cellular structures, including microspikes, filopodia, lamellipodia, and other actin-based protrusions underneath the plasma membrane.^3^ These structures are essential in cellular migration, cell-matrix adhesion, and cell-to-cell interactions. In healthy adult tissues, FSCN1 expression is restricted to the neuronal, endothelial, mesenchymal, and dendritic cells^4^ and is absent or at low levels in normal epithelial cells.^3^ FSCN1 has been shown to be unusually expressed in transformed epithelial cells and many human cancers. This implies that it may functionally contribute toward cancer progression.

Recently, FSCN1 has received a lot of attention, because multiple studies have implicated it as a candidate biomarker or therapeutic target for aggressive, metastatic carcinomas of many cancer types.^1^^,^^3^^,^^5^, ^6^, ^7^, ^8^, ^9^ Functional studies have revealed that FSCN1 promotes tumor cell migration, invasion, and metastasis.^1^^,^^8^^,^^10^ In addition, its overexpression is involved in epithelial-mesenchymal transition (EMT) that confers tumor cells with motility and invasive properties.^11^ Immunohistochemical (IHC) studies have demonstrated that FSCN1 protein expression is correlated with clinically aggressive phenotypes, poor prognosis, and survival outcomes.^1^^,^^5^^,^^11^^,^^12^ Systematic reviews and meta-analyses have revealed that FSCN1 is correlated with increased mortality risks and metastasis in various cancer types, is a novel biomarker for the identification of aggressive and metastatic tumors,^12^ and is a prognostic marker of overall survival.^13^ Targeted inhibition of FSCN1 functions with small molecule inhibitors blocking tumor cell migration, invasion, and metastasis.^7^^,^^8^^,^^14^^,^^15^ This shows the potential of FSCN1 as a therapeutic target.

In this study, we review the *FSCN1* gene and protein structure, its regulation of actin-bundling activity, and its physiological functions. The main focus was on the role of FSCN1 and its upregulatory mechanisms and significance in cancer cells. Up-to-date studies on FSCN1 as a novel biomarker and therapeutic target for human cancers are reviewed. It is shown that FSCN1 is an unusual biomarker and a potential therapeutic target for cancer.

### FSCN1 structure and activity regulation

#### FSCN1 gene and protein structure

**Figure 1:**
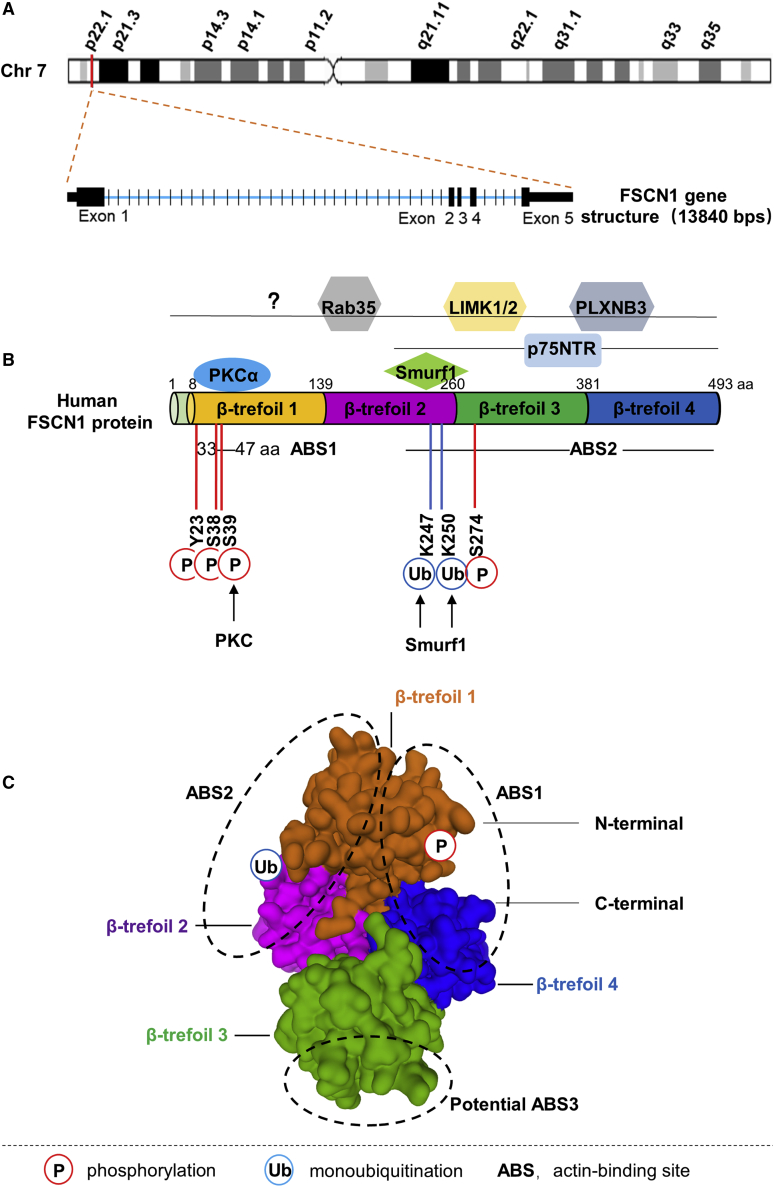
FSCN1 gene and protein structure, post-translational modifications, and interactions (A) Schematic of the FSCN1 gene that is located at chromosome 7p22.1 and is about 13.84 kb long, containing 5 exons (represented in black blocks). (B) Schematic diagram for human FSCN1 protein structure, post-translational modifications, and interactions. FSCN1 consists of four highly conserved β-trefoil domains. Actin-binding site 1 (ABS1) is located at the amino terminus, in the β-trefoil 1 domain between amino acids (aa) 33 and 47, whereas the ABS2 is predicted to locate at the carboxyl terminus, in the region near serine 274 (S274). Post-translational modification sites of FSCN1 are indicated below the FSCN1 structure: P, phosphorylation (in red) and S39 (in C); Ub, monoubiquitination (in blue) and lysine (K)247 and K250 (in C). FSCN1-interacting proteins that regulate its activity or function are represented above FSCN1 at their described binding site. (C) Surface presentation of the human FSCN1 (PDB: 3P53). The four β-trefoil domains are highlighted with different colors. Three ABSs (ABS1−3), identified from systematic mutagenesis studies, are also shown.

The human *FSCN1* gene (GenBank: NM_003088.4) is located on chromosome 7p22.1, containing 5 exons, 13,840 bp in length (Figure 1A). The orthologs of the human *FSCN1* gene are found in 224 organisms, and the *FSCN1* gene is conserved in chimpanzee, dog, cow, mouse, rat, chicken, zebrafish, and frog. The nucleotide sequence of the human *FSCN1* gene is highly homologous with mouse (96.55%) and zebrafish (75.76%), suggesting that *FSCN1* is likely to have fundamentally critical biological functions.

The human FSCN1 protein (GenBank: NP_003079.1) is a 493-amino acid (aa)-long protein with a molecular mass of 54.5 kDa. It is comprised of four tandem β-trefoil domains (residues 8–139, 140–260, 261–381, and 382–493) (Figure 1B). The X-ray crystal structure of human FSCN1 revealed that the four β-trefoil domains are arranged as two skewed lobes, corresponding to β-trefoil 1 and 2 and β-trefoil 3 and 4, respectively.^16^, ^17^, ^18^ One actin-binding site (ABS) has been shown to be located at the β-trefoil 1 domain between aa 33 and 47 in human FSCN1,^16^^,^^19^ whereas the second ABS has not yet been fully mapped. However, its location has been postulated to be at the region near serine 274 (Ser274) in human FSCN1 (Figure 1B).^16^^,^^20^ Recent studies have uncovered that there are three ABSs (ABS1−3) on the three distinct surface areas of the FSCN1 molecule^17^^,^^21^ (Figure 1C). The ABS1 is formed by residues from the N and C termini of FSCN1 and includes the cleft formed by β-trefoil 1 and 4.^17^ This region includes a highly conserved site (Ser39) that can be phosphorylated by protein kinase C (PKC).^19^^,^^22^^,^^23^ The ABS2 contrasts to ABS1 and includes residues from β-trefoil 1 and 2. The ABS3 is a potential site that involves β-trefoil 3.^17^ Therefore, all four β-trefoil domains of FSCN1 are involved in actin bundling.

#### Regulation of FSCN1 activity

FSCN1 is an evolutionarily conserved actin-bundling protein, in which its primary function is to cross link actin microfilaments into tight, relatively rigid, parallel bundles.^24^ The actin-bundling activity of FSCN1 is fundamentally linked to its structure and is regulated by a variety of factors, such as post-translational modifications,^20^^,^^22^^,^^25^, ^26^, ^27^ interaction partners,^19^^,^^22^^,^^27^, ^28^, ^29^, ^30^, ^31^ and small molecule compounds.^7^^,^^8^^,^^14^^,^^32^, ^33^, ^34^

Post-translational modifications of FSCN1 regulate its actin-bundling activity and function.^22^^,^^25^, ^26^, ^27^ Yamakita et al.^25^ demonstrated that phosphorylation of human FSCN1 inhibits its actin-binding and -bundling activities. The best-characterized phosphorylation site of FSCN1 is a conserved serine, Ser39, at its ABS1 that can be phosphorylated by PKC (Figure 1B).^19^^,^^22^ Evidence shows that Ser39 is associated with phosphorylation-dependent regulation of FSCN1 actin binding.^20^^,^^22^^,^^26^^,^^35^ In addition to Ser39, multiple phosphorylation sites on FSCN1 are involved in its actin-bundling activity. Zanet et al.^20^ documented that Ser274 can be phosphorylated to modulate the actin-bundling capacity of FSCN1 in human cancer cells. These findings are consistent with the characterization of an actin-binding domain of FSCN1 located in the region surrounding Ser274.^18^ In addition, Zeng et al.^35^ reported that phosphorylation of FSCN1 at tyrosine 23 and Ser38 is important in cell migration and filopodia formation in esophageal squamous cancer cells. Therefore, the balance between FSCN1 phosphorylation/dephosphorylation is important in the regulation of its activity. The regulatory mechanisms for FSCN1 phosphorylation/dephosphorylation have not been established; however, Ser39 phosphorylation is regulated by PKC and neurotrophin nerve growth factor.

Monoubiquitination is a post-translational modification that regulates FSCN1 actin-bundling activity and dynamics. Lin et al.^27^ revealed that FSCN1 was monoubiquitinated at two lysine residues (Lys247 and Lys250) in its ABS2. The E3 ubiquitin ligase Smurf1, which has been reported to interact with FSCN1, is partially responsible for catalyzing FSCN1 monoubiquitination. Moreover, they also revealed that monoubiquitination at ABS2 inhibited FSCN1 bundling activity by introducing steric hindrance to interfere with the interactions between FSCN1 and actin filaments.^27^ In addition to phosphorylation and monoubiquitination, the functional roles of other FSCN1 modifications, such as acetylation and methylation, have not been established.

Multiple FSCN1 interaction partners, including PKCα, Smurf1, plexin-B3 (PLXNB3), p-Lin-11/Isl-1/Mec-3 (LIM) kinases 1 and 2 (LIMK1/2), p75 neurotrophin receptor (p75^NTR^), and Rab35, regulate its activity or function (Figure 1B).^19^^,^^22^^,^^27^, ^28^, ^29^, ^30^, ^31^ Anilkumar et al.^19^ revealed that FSCN1 is a substrate and a binding partner of PKCα. They also showed that FSCN1-PKCα interactions occur dynamically in migrating cells and are important in the regulation of actin-crosslinking activity of FSCN1.^19^ Smurf1 is a E3 ubiquitin ligase that is essential in regulating the monoubiquitination of FSCN1.^27^ FSCN1 interacts with PLXNB3, the known functional receptor of Sema5A to mediate Sema5A-induced FSCN1 phosphorylation and actin network remodeling.^30^ It has also been reported that FSCN1 and LIMK1/2 form a complex that regulates the interactions of FSCN1 with actin and the stability of filopodia.^31^ FSCN1 interaction with p75^NTR^ is fascinating. This is because it is involved in the direct recruitment of FSCN1 to the plasma membrane, where it is dephosphorylated at Ser39 by neurotrophin nerve growth factor.^28^ In addition, Zhang et al.^29^ revealed that Rab35 directly associates with fascin and regulates the assembly of actin filaments during filopodia formation in cultured cells by recruiting fascin as an effector protein. In addition to the above interaction partners, various proteins have been found to physiologically interact with FSCN1.^36^, ^37^, ^38^, ^39^, ^40^, ^41^ However, it has not been established if these interactions regulate the actin-bundling activity of FSCN1.

The role of FSCN1 in tumor cell migration and invasion has been correlated to its actin-bundling activity. Therefore, various small molecule compounds that target FSCN1 and inhibit its activity have been recently identified.^7^^,^^8^^,^^14^^,^^32^^,^^33^ Chen et al.^7^ demonstrated that the metastasis inhibitory small molecules (migrastatin analogs) inhibit FSCN1 activity by binding to one of its ABSs. With the use of high-throughput screening, Huang and coworkers^8^^,^^14^ screened chemical libraries and identified small molecule compounds that specifically inhibited FSCN1 from bundling actin. They revealed that the small molecule compound G2 inhibits the actin-bundling function of FSCN1 and blocks tumor cell migration, invasion, and metastasis. In addition, a series of thiazole derivatives has also been reported to inhibit metastatic cancer cell migration and invasion by interfering with the actin-bundling function of FSCN1.^32^^,^^33^ However, the mechanisms by which thiazole derivatives regulate FSCN1 activity have not been elucidated.

#### The physiological function of FSCN1

FSCN1 is an actin-bundling protein that organizes actin filaments into parallel bundles. Therefore, it is involved in a broad range of cellular physiological processes, including regulation of cell adhesion, motility, migration, and cellular interactions.^2^^,^^42^^,^^43^ In addition, FSCN1 regulates focal adhesion dynamics,^44^ cell migration and invasion,^36^^,^^38^^,^^45^ histone methylation and gene transcription,^46^ extracellular vesicle release,^47^ and cancer cell stemness,^38^^,^^48^^,^^49^ independently of its actin-bundling activity. We review the actin-dependent and -independent functions of FSCN1 in this study.

#### Actin-dependent functions

As an actin-bundling protein, FSCN1 bundles or cross links actin filaments through three binding sites and is involved in the formation, as well as in the stability, of a wide range of cellular protrusions.^17^^,^^50^, ^51^, ^52^ Many of these FSCN1-containing protrusions, such as microspikes, filopodia, and lamellipodia, are transient, dynamic, and functionally required for cell adhesions, interactions, motility, and migration.^2^^,^^42^^,^^43^ Adams,^53^^,^^54^ in 1995 and 1997, respectively, revealed that the assembly of fascin microspikes is of functional significance for cell adhesion to specific extracellular matrix macromolecules. By interacting with active PKC that phosphorylates it on Ser39 and inhibits FSCN1/actin binding, FSCN1 is involved in cell adhesion to the extracellular matrix.^19^ Additionally, it has been shown that FSCN1 regulates focal adhesion dynamics in a variety of cell types. This regulation is partially dependent on its canonical actin-bundling function.^44^^,^^55^, ^56^, ^57^ FSCN1-containing protrusions are also important in cell interactions. For example, in breast epithelial tumor cells, fascin spikes play a role in sensing and responding to the extracellular insulin-like growth factor 1 stimulation.^58^ In dendritic cells, the large FSCN1-containing dendrites mediate effective interactions and antigen presentation to T cells.^59^

FSCN1 has been reported to play an important role in the regulation of cell motility and migration, which function in normal embryonic development and tumor progression.^1^, ^2^, ^3^^,^^60^ FSCN1 controls a variety of critical cell motility processes, such as directed cell migration, neurite or growth cone extension, and dendrite formation during normal development.^60^, ^61^, ^62^, ^63^, ^64^ When FSCN1 is expressed in the above processes, its effects on actin-filament structures are responsible for the enhancement of cell motility. Furthermore, FSCN1 is also highly expressed in many types of tumors (reviewed in Machesky and Li^11^) and promotes tumor cell migration, invasion, and metastasis. This elevates mortality risks.^1^^,^^12^^,^^55^ Although novel actin-independent roles of FSCN1 have been discovered,^36^^,^^38^^,^^45^ they do promote tumor cell migration and invasion through the formation of filopodia and invadopodia.^11^^,^^17^^,^^42^^,^^52^

#### Actin-independent functions

In addition to bundling actin, FSCN1 has multiple actin-bundling-independent cellular functions. Villari et al.^44^ documented that FSCN1 controls focal adhesion dynamics and cell migration by directly binding to microtubule cytoskeleton. The disruption of the interactions between FSCN1 and microtubules enhances cellular adhesion stability and decreases cell migration^44^. Moreover, the stimulatory effect of FSCN1 hyperexpression on breast cancer cell metastasis is dependent on the enhancement of microtubule dynamics, not its actin-bundling activity.^45^ FSCN1 interacts with the nuclear envelope protein nesprin-2 through a direct, actin-independent mechanism. This interaction is important for nuclear deformation and movement during cell-invasive migration.^36^ Additionally, FSCN1 maintains or increases cancer cell stemness in melanoma and breast cancer stem cells (CSCs), independently of its actin-bundling activity.^38^^,^^48^^,^^49^

Recently, several novel actin-independent roles of FSCN1 have been reported. Saad et al.^46^ revealed that phosphorylated fascin 1 (pFascin) is primarily localized in the nucleus and regulates histone methylation and gene transcription. pFascin specifically interacts with the H3K4 methyltransferase core subunit RbBP5 form H3K4me3. Nuclear pFascin interactions with the RNA polymerase II complex elucidate on the role of fascin in transcription.^46^ Lin et al.^65^ showed that FSCN1 promotes lung cancer metastatic colonization by augmenting metabolic stress resistance and mitochondrial oxidative phosphorylation. An additional actin-independent role of FSCN1 is the control of extracellular vesicle release. Beghein et al.^47^ showed that FSCN1 regulates extracellular vesicle release presumably depending on its microtubule-regulating function, independently of its actin-bundling activity. Clancy et al.^66^ documented that coordinated regulation of intracellular FSCN1 distribution is important for tumor microvesicle release. In addition, Lam et al.^63^ have also documented an actin-independent role of fascin in border cell migration during *Drosophila* oogenesis. It regulates delamination during border cell migration by altering E-cadherin localization in the border cells.

In summary, FSCN1 is a multifunctional protein that plays an important role in regulating various cellular physiological processes in normal and tumor cells. Most of these processes, particularly tumor cell migration and metastasis, are attributed to its actin-dependent and actin-independent functions. However, it has not been established whether the two distinct functions of FSCN1 synergistically regulate migration and metastasis or not. Elucidation of FSCN1 regulatory mechanisms in certain cancer types is important for the development of targeted therapeutic agents.

### Role of FSCN1 in cancer

FSCN1 is associated with cell motility. In 2000, the first report on the analysis of FSCN1 in human breast cancer was published.^67^ It was shown that FSCN1 upregulation enhances the aggressiveness of human breast cancer. After that, a series of studies has shown that FSCN1 is highly expressed in different cancer types and that its expression is associated with aggressive clinical course, poor prognosis, and shorter survival outcomes.^1^^,^^3^^,^^9^^,^^11^^,^^12^ Functional studies using human cancer cell lines have shown that FSCN1 is involved in the regulation of cancer-related cellular properties, such as growth, migration, invasion, metastasis, and therapeutic resistance. The essential findings are summarized in Tables 1 and 2.

**Table 1:** The biological effects on downregulation of FSCN1 *in vitro* experiments

Cancer type	Cell lines	Proliferation	Growth	Migration and invasion	Metastasis	Drug resistance	Refs.
Bladder cancer	T24	inhibited	unknown	inhibited	unknown	unknown	^68^
	T24, BIU87	unknown	unknown	inhibited	unknown	unknown	^69^
	5637, BIU87	no effect	unknown	inhibited	unknown	unknown	^70^
Breast cancer	Bcap-37, HCC-1937, MDA-MB-468, MDA-MB-231	unknown	unknown	inhibited	unknown	unknown	^71^^,^^72^
	MCF-7, MDA-MB-435, MDA-MB-231	inhibited	unknown	inhibited	unknown	unknown	^73^^,^^74^
	MDA-MB-231	unknown	unknown	unknown	unknown	decrease resistance to doxorubicin	^75^
	MDA-MB-231	no effect	unknown	inhibited	unknown	unknown	^76^
Cervical cancer	HeLa	inhibited	unknown	unknown	unknown	unknown	^77^
	CaSki	inhibited	inhibited	inhibited	unknown	unknown	^78^
Cholangiocarcinoma	QBC939	inhibited	inhibited	unknown	unknown	unknown	^79^
Chondrosarcoma	JJ012, SW1353	no effect	unknown	inhibited	unknown	unknown	^80^
Colorectal cancer	SW480Pa, IKD-F11	unknown	inhibited	inhibited	inhibited	unknown	^55^
	SW480, HCT116, SW620, LoVo, HT-29	inhibited	unknown	inhibited	unknown	unknown	81, 82, 83, 84
	SW480	unknown	unknown	inhibited	unknown	unknown	^85^
Esophageal cancer	KYSE 170	inhibited	inhibited	unknown	unknown	unknown	^86^
	KYSE 150, T.Tn, TE2	inhibited	unknown	inhibited	unknown	unknown	^87^^,^^88^
	KYSE-30	inhibited	inhibited	inhibited	unknown	unknown	^89^
Gastric cancer	SGC-7901	inhibited	unknown	unknown	unknown	unknown	^90^
	MGC-803, SGC-7901	unknown	unknown	inhibited	unknown	unknown	^91^
	HGC-27, MGC-803, MKN-28, AGS	inhibited	unknown	inhibited	unknown	unknown	92, 93, 94
	MKN45	inhibited	unknown	inhibited	inhibit liver metastasis	unknown	^95^
	MKN45	unknown	unknown	inhibited	inhibited	unknown	^96^
	SGC-7901, MKN45	unknown	inhibited	inhibited	unknown	unknown	^97^
Glioma	U251, U87, SNB19	unknown	unknown	inhibited	unknown	unknown	^98^
Hepatocellular carcinoma	SNU449, SNU387, Huh7, Hep3B	inhibited	unknown	unknown	unknown	decrease resistance to doxorubicin	^99^
	HLE	no effect	unknown	inhibited	unknown	unknown	^100^
Laryngeal cancer	Hep-2, TU-177	inhibited	inhibited	inhibited	unknown	unknown	^101^
Lung cancer	SPC-A1, H1299	inhibited	unknown	inhibited	unknown	decrease resistance to docetaxel	^102^
	H1650	no effect	unknown	unknown	unknown	unknown	^65^
	H292	unknown	unknown	unknown	inhibited	unknown	^65^
Melanoma	BLM, FM3P, WM793	no effect	unknown	no-effect cell migration; enhance cell invasion	unknown	unknown	^103^
Nasopharyngeal carcinoma	SUNE-1, CNE-2, 5-8F, CNE1	unknown	unknown	inhibited	unknown	unknown	^104^^,^^105^
	SUNE-1, CNE-2	inhibited	unknown	inhibited	unknown	unknown	^106^
Non-small cell lung cancer	A549, H520, SPC-A-1	inhibited	unknown	inhibited	unknown	unknown	^107^^,^^108^
	H1229, H129	unknown	unknown	inhibited	unknown	unknown	^109^^,^^110^
Oral cancer	HSC-3, SCC-15	no effect	inhibited	inhibited	unknown	unknown	^111^
	OEC-M1, SCC-25	unknown	unknown	inhibited	unknown	unknown	^112^
Ovarian cancer	SKOV3, 3AO	unknown	unknown	inhibited	unknown	unknown	^39^^,^^113^
	SKOV3, OVCAR3	inhibited	unknown	inhibited	unknown	unknown	^114^
	HeyA8, Ovcar5, Tyk-nu	unknown	unknown	inhibited	inhibited	unknown	^115^
Pancreatic cancer	MIA PaCa-2	unknown	unknown	inhibited	unknown	unknown	^116^
Prostate cancer	PC3, DU145	inhibited	unknown	inhibited	unknown	unknown	^117^
	DU145	unknown	inhibited	inhibited	inhibit lymph node metastasis	unknown	^10^
Renal cancer	769-P, OSRC, 786-0	unknown	unknown	inhibited	unknown	unknown	^118^^,^^119^
	A498, 786-O	unknown	unknown	inhibited	inhibit lung metastasis	unknown	^120^
Tongue cancer	CAL-27, SCC-25	inhibited	inhibited	inhibited	unknown	unknown	^121^

**Table 2:** The biological effects on upregulation of FSCN1 *in vitro* experiments

Cancer type	Cell lines	Proliferation	Growth	Migration and invasion	Metastasis	Drug resistance	Refs.
Breast cancer	MDA-MB-468, MDA-MB-231	unknown	unknown	enhanced	unknown	unknown	^71^^,^^122^
	MDA-MB-231	no effect	no effect	no effect	enhance lung metastasis	unknown	^45^
	MDA-MB-231	no effect	unknown	enhanced	unknown	unknown	^76^
	T47-D, SK-BR-3	unknown	unknown	unknown	unknown	increase resistance to doxorubicin	^75^
	MDA-MB-435, MDA-MB-231	enhanced	unknown	enhanced	unknown	unknown	^73^
Cholangiocarcinoma	RBE	enhanced	unknown	enhanced	unknown	unknown	^123^
Colorectal cancer	AAC1, ANC1, RGC2, HT-29, SW620, LoVo	unknown	unknown	enhanced	unknown	unknown	^82^^,^^124^
	HT-29	unknown	unknown	enhanced	enhance lung metastasis	unknown	^81^
Esophageal cancer	SHEE	enhanced	unknown	enhanced	unknown	unknown	^88^
Hepatocellular carcinoma	Huh7, SK-HEP1	unknown	unknown	enhanced	unknown	unknown	^100^^,^^125^
Hypopharyngeal cancer	FaDu	unknown	unknown	enhanced	unknown	unknown	^126^
Lung cancer	SPC-A1, H1299	enhanced	unknown	enhanced	unknown	increase resistance to docetaxel	^102^
Non-small cell lung cancer	A549, SPC-A-1	enhanced	unknown	enhanced	unknown	unknown	^107^
	A549	no effect	no effect	enhanced	enhanced	unknown	^127^
Oral cancer	AW13516	enhanced	enhanced	enhanced	unknown	unknown	^128^
Osteosarcoma	SaOS-2, 143B	unknown	enhanced	enhanced	enhance lung metastasis	unknown	^129^
Ovarian cancer	SKOV3, 3AO	unknown	unknown	enhanced	unknown	unknown	^39^
Pancreatic cancer	MIA PaCa-2	no effect	unknown	enhanced	enhance skin metastasis	unknown	^130^
	MIA PaCa-2	unknown	unknown	enhanced	unknown	unknown	^116^
Prostate cancer	PC3, DU145	unknown	unknown	unknown	unknown	increase resistance to paclitaxel	^131^

#### Upregulation of FSCN1 expression in cancer

**Figure 2:**
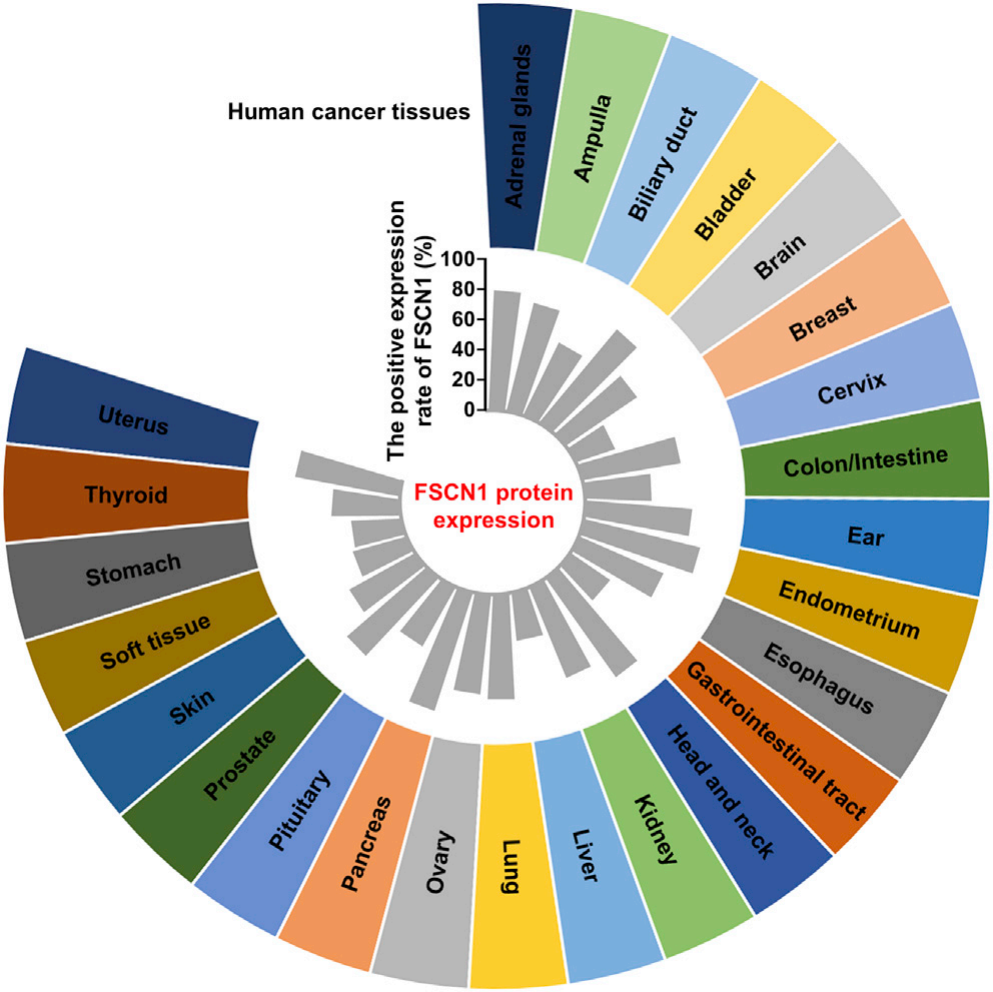
Expression of FSCN1 protein in different human cancer tissues The average positive rate of FSCN1 protein expression in different types of human cancer tissues is shown in the center of the figure.

The absence or low expression of FSCN1 in normal epithelia is altered in different human carcinomas. To date, more than 100 studies were published on FSCN1 expression in tumors and malignancies from various tissues and organs examined by IHC, tissue microarray, qPCR, or western blot analysis (summarized in Table S1). In nearly all cancers, the positive expression rate of FSCN1 was increased in cancer tissues compared to that in normal epithelium or para-carcinoma tissues. Evidence shows that there are tissue-specific mechanisms that regulate FSCN1 expression. This explains why the various proportions of FSCN1 in FSCN1-positive tumors, detected in different human tissues, vary significantly (Figure 2). For example, more than 80% of bladder, as well as head and neck cancer tissues, express high levels of FSCN1. However, in breast or gastric cancer tissues, the average frequency of FSCN1 upregulation is 27% and 33%, respectively (Figure 2; Table S1). Pelosi et al.^132^ showed that FSCN1 immunoreactivity was detected in 5% of typical carcinoids, 35% of atypical carcinoids, 83% of large-cell neuroendocrine carcinomas, and 100% of small-cell lung carcinomas, even though they are all lung tumors. The frequency of FSCN1-positive expression has been established to be higher in most clinically aggressive tumors. In breast and kidney cancer, the frequency of FSCN1-positive tumors tended to increase with tumor invasion and metastasis.^71^^,^^133^ In addition, in the squamous cell carcinoma, especially head and neck squamous cell carcinoma, FSCN1 upregulation is frequently high (Table S1).

#### FSCN1 upregulation mechanisms in cancer

Several studies have been aimed at understanding the molecular basis for elevated FSCN1 protein expression levels in human cancers. The regulation of FSCN1 expression in cancers is challenging and is affected by both transcriptional and post-transcriptional mechanisms. Studies have also shown that alterations in gene copy numbers are not associated with FSCN1 upregulation.

**Figure 3:**
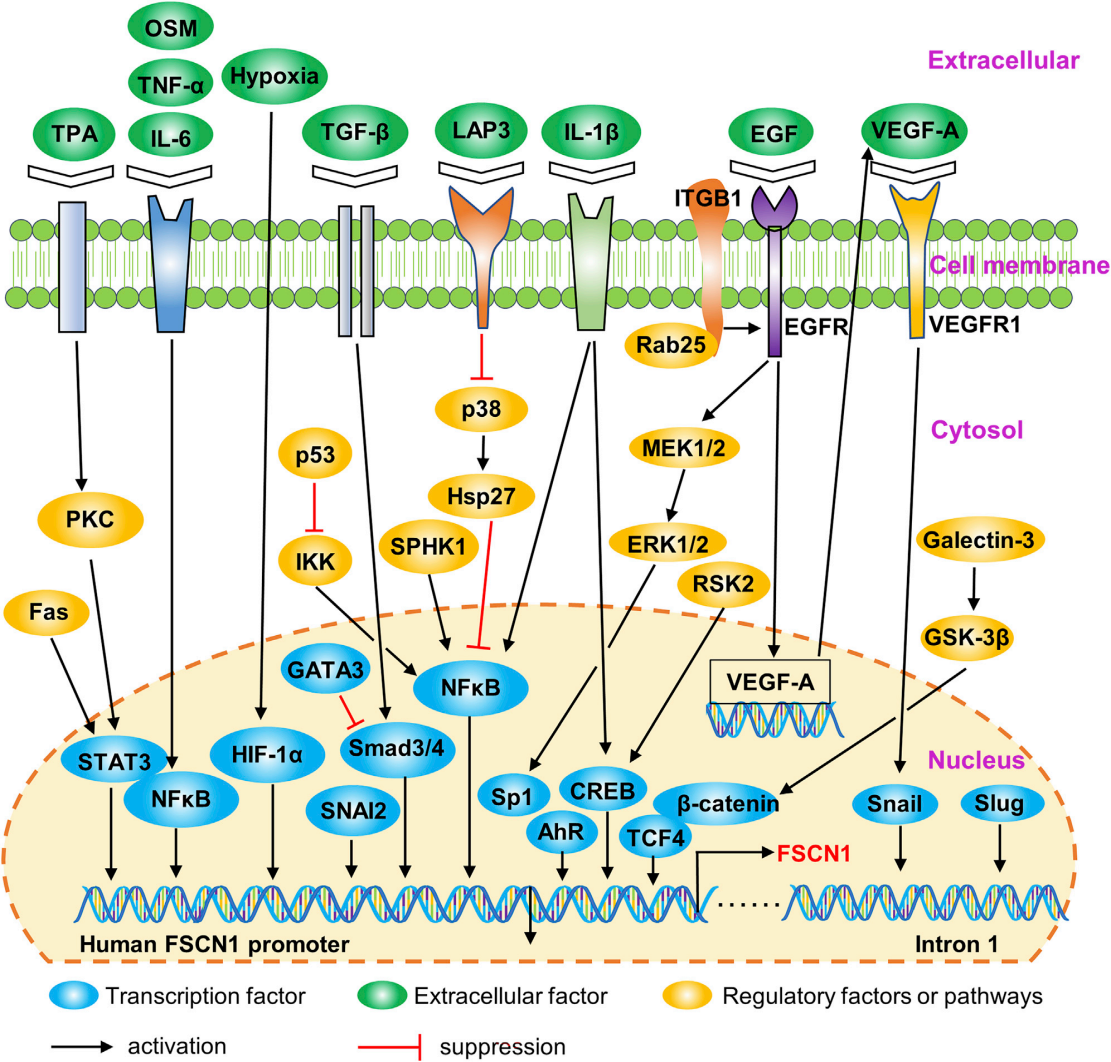
Transcriptional regulation of FSCN1 in human cancer Multiple transcriptional factors bind to the promoter regions of the *FSCN1* gene. Regulatory factors or signaling pathways that regulate FSCN1 expression by activating the transcriptional factors are also shown.

*Transcriptional factors (TFs) and related signaling pathways*. Mechanisms of transcriptional regulation for FSCN1 in human cancers have been studied. As shown in Figure 3, multiple TFs bind *FSCN1* gene promoter regions and play an important role in regulating FSCN1 transcription. Different regulatory factors and signaling pathways regulate FSCN1 expression by activating TFs in different cancer cell types (Figure 3).

FSCN1 is regulated by the cyclic AMP (cAMP) response element-binding protein (CREB)-binding protein in NT2 neuronal precursor cells.^134^ Hashimoto et al.^135^ documented that the transcriptional activity of FSCN1 is regulated by a promoter region (−219/+114).

They also showed that CREB and aryl hydrocarbon receptors (AhRs) specifically associate with the −219/+114 region of the *FSCN1* promoter in FSCN1-positive human breast and colon cancer cells. Li et al.^136^ found that the expression of FSCN1 can be suppressed by p90 ribosomal S6 kinase 2 (RSK2) knockdown in cell lines from diverse human cancers in a CREB-dependent manner. A recent study established that the CREB signaling pathway is involved in interleukin (IL)-1β-induced FSCN1 expression in human oral cancer cells.^137^

In addition, the β-catenin-T cell factor (TCF; TF) signaling pathway is involved in the regulation of FSCN1 transcription in human colorectal cancer cells,^81^ whereas galectin-3 enhances the expression of FSCN1 in human gastric cancer cells by regulating the glycogen synthase kinase 3 (GSK)-3β/β-catenin/TCF-4 signaling pathway.^92^ However, some studies do not support the hypothesis that the β-catenin-TCF pathway has a specific role in regulating FSCN1 transcriptional activity in human MDA-MB-435 cells or in fascin-positive human colon cancer cells.^135^^,^^138^ It has not been established whether the expression of FSCN1 in human cancers is regulated by β-catenin-TCF signaling.

FSCN1 is specifically regulated by TF specificity protein 1 (Sp1) in esophageal squamous cell carcinoma (ESCC).^139^ Sp1 upregulates FSCN1 transcription by binding to the key element located at −70 to −60 nt of the FSCN1 promoter. It was also observed that stimulation with epidermal growth factor (EGF) enhanced FSCN1 expression. This inductive effect exerted by EGF was found to be dependent on the activation of the mitogen-activated protein kinase (MAPK) kinase (MEK)-extracellular signal-regulated kinase 1/2 (ERK1/2)-Sp1 signaling pathway.^139^

Signal transducer and activator of transcription 3 (STAT3) and nuclear factor κB (NF-κB) are two well-known TFs required for the cytokine-induced expression of FSCN1 in human cancer cells.^140^, ^141^, ^142^, ^143^, ^144^ FSCN1 expression is induced by a variety of cytokines, such as IL-6, tumor necrosis factor (TNF)-α, and oncostatin M (OSM). These cytokines activate STAT3 and NF-κB in human breast and gastric cancer cells to induce the expression.^140^, ^141^, ^142^ A 160-bp conserved region of the FSCN1 promoter has been shown to contain overlapping STAT3 and NF-κB sites.^140^ STAT3 and NF-κB are co-dependent in their binding to the FSCN1 promoter in response to cytokine treatment.^140^, ^141^, ^142^ In addition to cytokines, several signaling pathways regulate FSCN1 expression by activating STAT3 or NF-κB. Yang et al.^145^ revealed that Fas signaling promotes FSCN1 expression by activating STAT3 in AGS gastric cancer cells. 12-O-tetradecanoylphorbol 13-acetate (TPA)-induced FSCN1 gene transcription is partially mediated by the PKCδ/STAT3α signaling pathway in MCF-7 breast cancer cells.^144^ In addition, p53 suppresses FSCN1 expression by inhibiting NF-κB signals in colorectal cancer cells.^146^ Fang et al.^147^ reported that leucine aminopeptidase 3 promotes FSCN1 expression through the p38-Hsp27-NF-κB signaling pathway. It has also been reported that FSCN1, in triple-negative breast cancer, is transcriptionally upregulated by sphingosine kinase 1, which activates NF-κB.^148^

The expression of FSCN1 can be enhanced by transforming growth factor-beta (TGF-β) treatment in spindle-shaped tumor cells in a Smad-dependent manner.^149^ Depletion of TF Smad3 or Smad4 by short hairpin (sh)RNA was shown to inhibit TGF-β-induced FSCN1 expression in human MDA-MB-231 cells and A549 cells.^149^ The luciferase reporter assay demonstrated that the Smad4 transcription complex promoted FSCN1 expression by directly binding to the −370 CAGAC site of the FSCN1 promoter.^150^ In breast cancer cells, GATA3 inhibits Smad4-mediated FSCN1 overexpression by suppressing the binding of Smad4 to the FSCN1 promoter.^150^ In gastric cancer, the induction of FSCN1 expression by TGF-β is partially dependent on Smad3 linker phosphorylation.^151^

Hypoxia-inducible factor-1α (HIF-1α) is a TF that has been implicated in FSCN1 expression. It is required for hypoxia-induced overexpression of FSCN1 in pancreatic and hypopharyngeal cancer cells.^116^^,^^126^ HIF-1α knockdown by specific small interfering RNA (siRNA) was shown to suppress the expression of FSCN1 under hypoxia.^116^^,^^126^ Chromatin immunoprecipitation analysis revealed that FSCN1 is a direct target gene for HIF-1α. Hypoxic microenvironments upregulate FSCN1 expression by enhancing the binding of HIF-1α to a hypoxia response element on the FSCN1 promoter and transactivating FSCN1 transcription.^116^

Evidence suggests that snail family TFs are involved in the regulation of FSCN1 transcription. Li et al.^152^ reported that the expression of snail or slug (also called snail2) in human pancreatic cancer cells PANC-1 and human colon cancer cells HT29 induced FSCN1 expression. They found a conserved slug-binding E-box sequence located within the first intron of the mammalian fascins. slug co-precipitated with this putative fascin E-box element in mouse pancreatic cancer cells.^152^ Wang et al.^153^ documented that snail family transcriptional repressor 2 (SNAI2) elevates the expression of FSCN1 at both mRNA and protein levels in head and neck cancer cells by binding the FSCN1 promoter. In addition, Jeong et al.^154^ demonstrated that a integrin β1 (ITGB1)/EGF receptor (EGFR)/vascular endothelial growth factor (VEGF)-A/VEGF receptor (VEGFR)-1/snail signaling axis is critical for Rab25-induced cancer cell aggressiveness through induction of FSCN1 expression.

**Figure 4:**
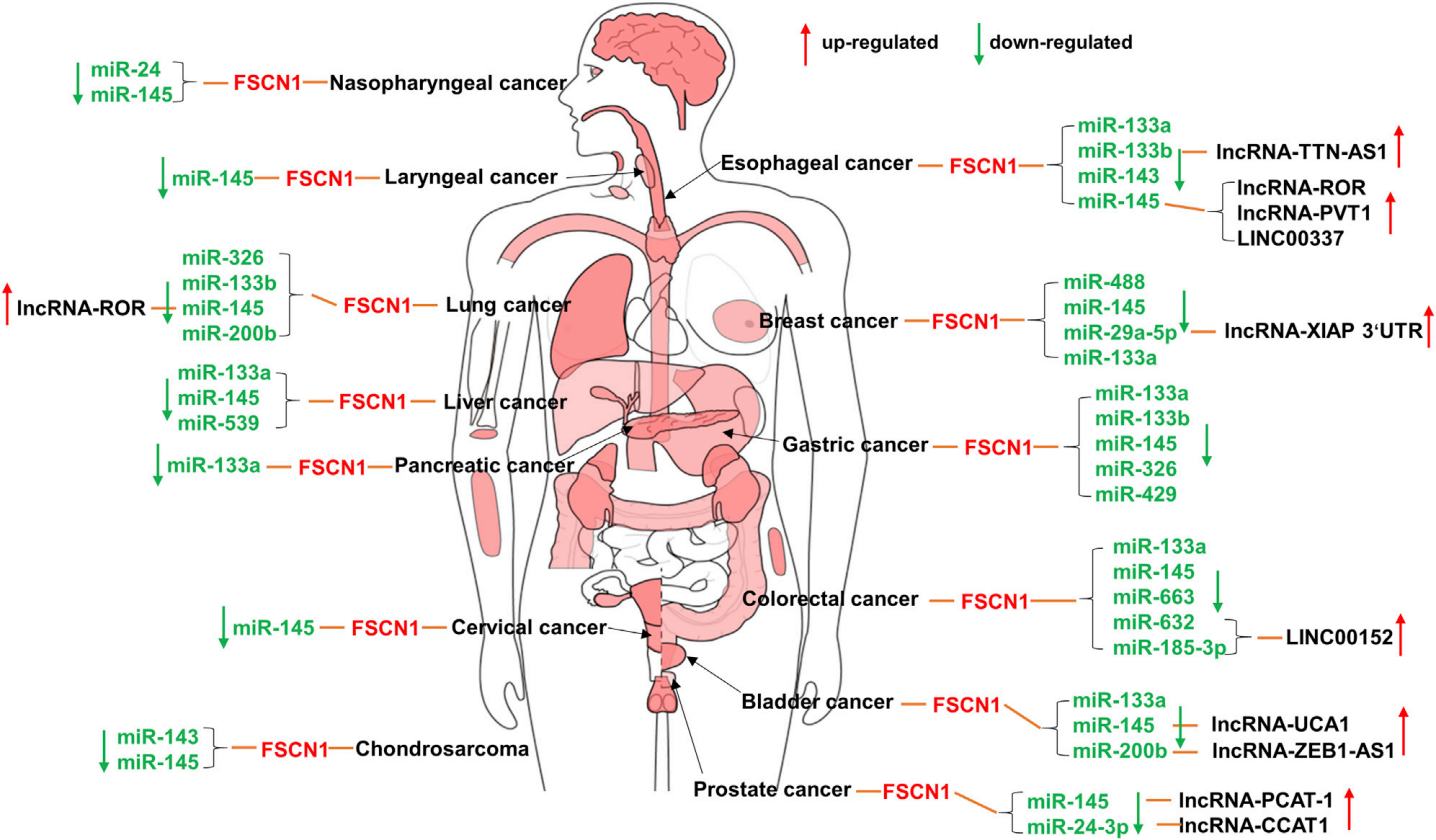
Regulation of FSCN1 expression by miRNAs and long-noncoding RNAs in different human cancers The body map of FSCN1 expression in tumors reproduced from Gene Expression Profiling Interactive Analysis (GEPIA).^155^

*MicroRNAs (miRNAs and miRs) and long-noncoding RNAs (lncRNAs)*. Many miRNAs bind the 3′ UTR of the FSCN1 transcript and lead to negative regulation. As shown in Figure 4, miR-145 negatively regulates FSCN1 mRNA levels in many different human cancers, including nasopharyngeal, laryngeal, esophageal, breast, lung, stomach, liver, colon, bladder, cervix, prostate, and cartilage cancers.^72^^,^^77^^,^^80^^,^^82^^,^^101^^,^^104^^,^^109^^,^^117^^,^^156^, ^157^, ^158^, ^159^ miR-133a/b directly targets FSCN1 in a variety of human cancers and acts as a tumor suppressor.^87^^,^^93^^,^^158^^,^^160^, ^161^, ^162^, ^163^, ^164^ Furthermore, the *FSCN1* gene has been identified as a direct target of several miRNAs, such as miR-143 in chondrosarcoma and esophageal carcinoma,^80^^,^^165^ miR-24 in nasopharyngeal and prostate cancer,^106^^,^^131^ miR-326 in lung and gastric cancer,^166^^,^^167^ and so on (summarized in Figure 4). All of the above-mentioned miRNAs were, however, significantly downregulated in corresponding human cancer specimens or cell lines and exerted their anti-tumor functions through a coordinated regulation of FSCN1.

Cancer-associated lncRNAs compete endogenous RNA (ceRNA) in the regulation of FSCN1 expression through miRNAs. Xue and colleagues^159^ reported that lncRNA-UCA1 mediates bladder cancer progression through the miR-145-FSCN1 pathway. Different studies have confirmed this finding. As shown in Figure 4, the functions of lncRNA-regulatory of reprogramming (ROR) in lung and esophageal cancer depend on the sponging of miR-145, thereby upregulating the expression of FSCN1.^102^^,^^156^ Moreover, lncRNA-PVT1, lncRNA-PCAT-1 and long intergenic noncoding RNA (LINC)00337 also act as ceRNA of miR-145 in esophageal and prostate cancer.^168^, ^169^, ^170^ In addition, lncRNA-TTN-AS1 enhances FSCN1 expression by competitively binding to miR-133b in esophageal cancer.^160^ Wu et al.^171^ showed that lncRNA-XIAP-3′ UTR antagonizes miR-29a-5p, resulting in the increased translation of FSCN1 in breast cancer. Gao et al.^172^ found that lncRNA-ZEB1-AS1 functions as a ceRNA in bladder cancer and regulates the expression of FSCN1 through miR-200b. It has recently been established that LINC00152 regulates FSCN1 by sponging with miR-632 and miR-185-3p in colorectal cancer.^173^ lncRNA-CCAT1 interference contributes to the sensitivity of paclitaxel by regulating miR-24-3p and FSCN1.^131^ With the advances in high-throughput RNA deep sequencing, it is postulated that additional lncRNAs involved in regulating FSCN1 will be revealed.

Two miRNAs (miR-146a and miR-451) do not regulate FSCN1 expression by binding the 3′ UTR of the FSCN1 transcript. Kanda et al.^174^ revealed that stable FSCN1 expression in colon adenocarcinoma cells is induced by the inhibition of proteasome degradation by miR-146a. Chen et al.^83^ found that miR-451 was overexpressed in colorectal cancer cells, thereby inhibiting AMP-activated protein kinase (AMPK) from activating mammalian target of rapamycin (mTOR) complex 1 (mTORC1) that promotes FSCN1 expression.

The above-discussed molecular mechanisms are known for enhancing FSCN1 expression by cancer cells. There are multiple mechanisms for FSCN1 upregulation in human cancers. Although the upregulation of FSCN1 has been extensively studied in many different cancer cells—several regulatory factors or signaling pathways regulate FSCN1 expression—their underlying mechanisms have not been established. Therefore, integrated studies of these mechanisms in same cell types are needed to better understand the complex regulation of FSCN1 expression.

#### Functional consequences of FSCN1 upregulation in cancer cells

**Figure 5:**
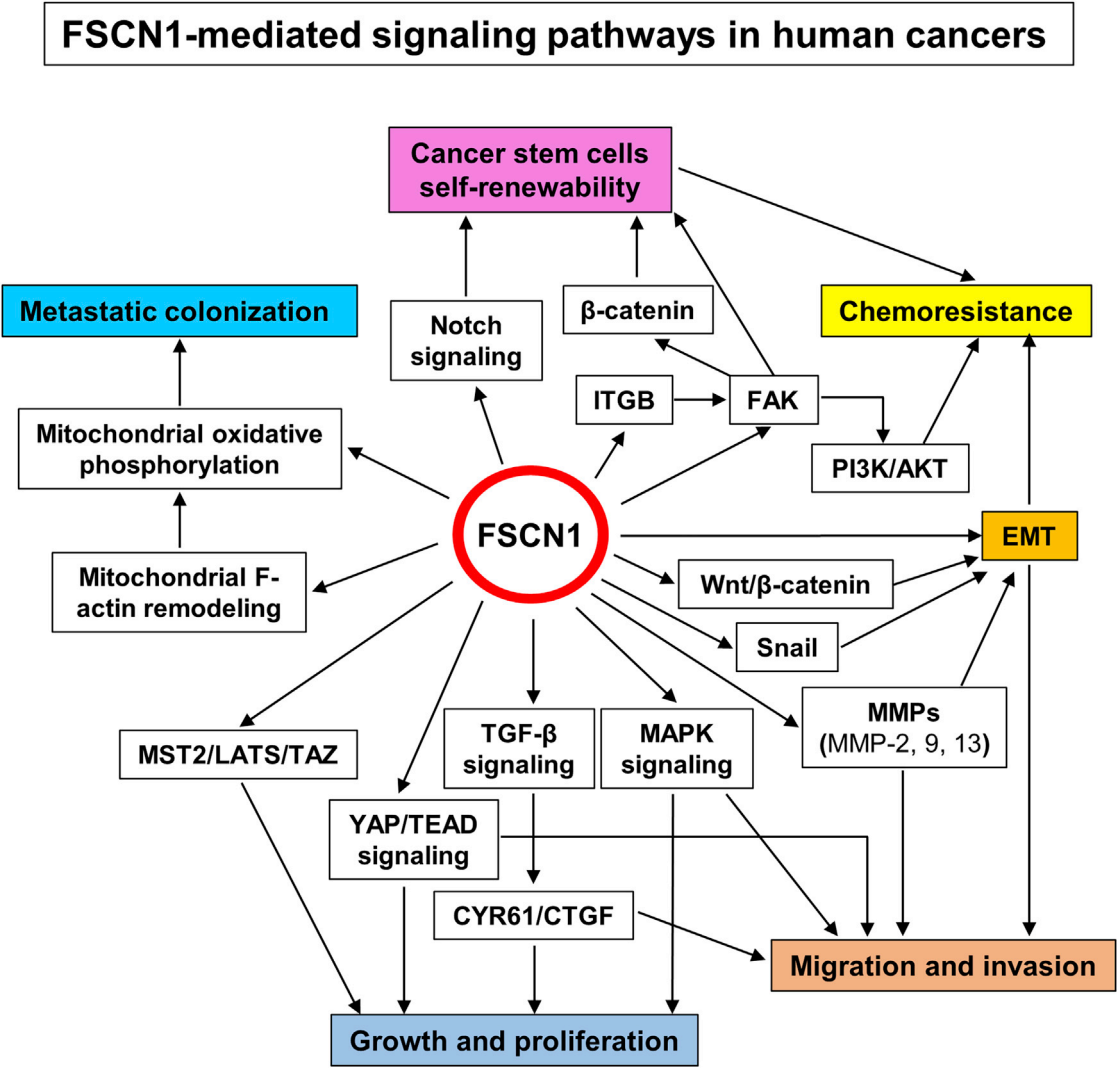
Schematic representation of the known signaling pathways mediated by FSCN1 in human cancers

FSCN1 is highly upregulated, both at the mRNA and protein level, in various human cancer cell lines. How does this expression associate functionally with malignant properties of cancer cells? *In vitro* and *in vivo* studies have been performed in cancer cell lines using FSCN1 depletion or overexpression. Various cancer-related cellular properties, such as growth, migration, invasion, metastasis, and drug resistance, are affected by the forced changes in FSCN1 expression (summarized in Tables 1 and 2). It is not known if FSCN1 affects these features in a direct or indirect fashion. This is associated with the different types of FSCN1-mediated signaling pathways that are involved in the malignant properties of cancer cells (Figure 5).

*Cell proliferation and tumor growth*. With respect to imbalanced tumor growth, contrasting results have been reported upon *in vitro* manipulations of FSCN1 expression levels in cell lines. The decrease of the level of FSCN1 using synthetic siRNA or vector-based shRNA inhibits *in vitro* cell growth in many human cancer cell lines (Table 1). The overexpression of FSCN1 in breast (MDA-MB-435 and MDA-MB-231),^73^ cholangiocarcinoma (REB),^123^ esophageal (SHEE),^88^ lung (SPC-A-1, H1229),^102^^,^^107^ or oral (AW13516)^128^ cancer cell lines was found to enhance cell proliferation rates (Table 2). However, FSCN1 knockdown did not exhibit any effect on cell proliferation in multiple cancer cell lines, including bladder cancer (5637 and BIU87),^70^ chondrosarcoma (JJ012 and SW1353),^80^ hepatocellular carcinoma (HLE),^100^ lung cancer (H1650),^65^ melanoma (BLM, FM3P, and WM793),^103^ and oral cancer (SSC-15 and HSC-3)^111^ cell lines. Heterogeneous expression of FSCN1 also does not promote pancreatic cancer (MIA PaCa-2 cell line) cell proliferation.^130^

The contrasting roles of FSCN1 in cell proliferation have also been reported in lung cancer cell A549^107^^,^^108^^,^^127^ and breast cancer cell MDA-MB-231^45^^,^^73^^,^^76^ lines. Zhao et al.^127^ showed that upregulated FSCN1 exhibited a light influence on A549 non-small cell lung cancer (NSCLC) cell proliferation. However, Liang et al.^107^ found that FSCN1 enhances A549 cell proliferation by activating the YAP/TEAD signaling pathway. Moreover, Zhao et al.^108^ documented that FSCN1 knockdown suppresses NSCLC (A549 cells) proliferation and tumor growth through the MAPK signaling pathway. These findings were also observed in breast cancer cells. Xing et al.^73^ revealed that forced expression of FSCN1 promoted MDA-MB-231 breast cancer cell proliferation, whereas FSCN1 knockdown inhibited MDA-MB-231 cell proliferation. Contrastingly, Al-Alwan et al.^76^ claimed that FSCN1 expression did not exhibit any effect on MDA-MB-231 cell proliferation. Furthermore, Heinz et al.^45^ also revealed that hyperexpression of FSCN1 in MDA-MB-231 cells did not exhibit any effect on cell proliferation. They hypothesized that this could be implicated in the concentration of FSCN1 protein, as there was a high basal FSCN1 level in MDA-MB-231 cells.^45^

In addition, injection of cancer cells, stably knocked down FSCN1 in nude mice, showed a suppressed tumor growth rate compared to control cells (Table 1). The tumor growth rate of nude mice administered with FSCN1-overexpressed cells was shown to be faster compared to the control cells of oral cancer (AW13516) and osteosarcoma (SaOS-2 and 143B).^128^^,^^129^ However, contrasting results have been reported. For example, Zhao et al.^127^ revealed that FSCN1 overexpression in A549 NSCLC cells exhibited a limited effect on tumor growth, whereas Heinz et al.^45^ showed that the hyperexpression of FSCN1 in MDA-MB-231 breast cancer cells did not exhibit any effect on tumor growth in xenograft mouse models.

Studies have reported that manipulation of FSCN1 expression in cancer cells affects tumor cell growth and proliferation. However, only a few of these studies analyzed the underlying mechanisms of action (Figure 5). For example, Xie et al.^88^ documented that FSCN1 regulates the proliferation of ESCC cells by modulating the expression of CTGF and CYR61 through the TGF-β signaling pathway. The MAPK and YAP/TEAD signaling pathways are involved in the FSCN1-mediated growth, migration, and invasion of NSCLC cells.^107^^,^^108^ Finally, Kang et al.^38^ demonstrated that the MST2-LATS-TAZ pathway plays an important role in FSCN1-induced melanoma tumorigenesis.

*Migration, invasion, and metastasis*. Downregulation of FSCN1, in a variety of cancer cell lines, not only inhibited their proliferative capacity but also their migratory and invasive abilities (Table 1). This reduction in migratory and invasive abilities was accompanied by an inhibited EMT process.^79^^,^^97^^,^^101^ On the contrary, the overexpression of FSCN1 was shown to promote the capacity for cell migration and invasion in many cancer cells, such as hypopharyngeal cancer cells (FaDu),^126^ osteosarcoma cells (SaOS-2 and 143B),^129^ and pancreatic cancer cells (MIA PaCa-2),^116^ among others (Table 2). However, in melanoma, FSCN1 knockdown did not exhibit inhibitory effects on cell migration but enhanced cell invasion.^103^ Studies have established an inverse relationship between FSCN1 expression and malignant or metastatic melanomas.^175^, ^176^, ^177^ Therefore, it is clear that FSCN1 plays a different role in melanoma compared to the other tumor types. In addition, the hyperexpression of FSCN1 was shown not to exhibit any significant effects on transmigration of MDA-MB-231 breast cancer cells.^45^ This finding is not in tandem with findings from other studies.^71^^,^^73^^,^^76^^,^^122^ More studies are therefore needed to understand the roles of FSCN1 in breast cancer progression.

Forced changes in FSCN1 expression were shown to alter cell metastasis *in vivo*. To evaluate the effects of FSCN1 on tumor spreading in a physiologically faithful microenvironment, several studies performed orthotopic transplantation of cancer cell lines into the corresponding organs of athymic nude mice.^10^^,^^55^^,^^120^^,^^129^ Downregulation of FSCN1 expression in prostate cancer cells DU145^10^ or renal cancer cells 786-O^120^ was shown to suppress tumor metastases in nude mice, whereas the overexpression of FSCN1 in osteosarcoma SaOS-2 cells enhanced lung metastasis in severe combined immunodeficiency (SCID) mice. The injection of cancer cells with FSCN1 knockdown into the abdominal cavities of nude mice suppressed metastatic tumor nodes when compared to those injected with control cells.^95^^,^^96^^,^^115^ Furthermore, FSCN1 overexpression enhances the metastasis of cancer cells.^45^^,^^65^^,^^81^^,^^127^^,^^130^ Upregulated FSCN1 in human tumors is functionally involved in tumor cell migration, invasion, and metastasis.

FSCN1 is an actin-bundling protein that cross links actin filaments to promote cell migration and invasion by forming stable filopodia and invadopodia.^1^^,^^31^^,^^52^ Several studies have also documented that targeted inhibition of FSCN1/actin bundling blocks tumor cell migration and metastasis.^7^^,^^8^^,^^14^ Moreover, Lin et al.^65^ reported that FSCN1 plays a role in regulating lung cancer metastatic colonization and mitochondrial oxidative phosphorylation by remodeling mitochondrial actin filaments (Figure 5).

The significant functions of FSCN1 in tumor cell migration, invasion, and metastasis depend on its role in actin cytoskeletal reorganization and on the activities of FSCN1-mediated cell signaling pathways. EMT is a cellular process during which epithelial cells lose their epithelial characteristics and acquire a migratory and invasive mesenchymal phenotype. It is established that EMT is activated during cancer pathogenesis and is involved in enhancing migration, invasion, and metastasis.^178^ Evidence shows that FSCN1 is involved in the EMT process in a number of cancer types, including squamous cell carcinoma,^101^^,^^111^^,^^112^^,^^153^ cholangiocarcinoma,^79^ gastric cancer,^97^ and ovarian cancer.^113^ Varying the expression levels of FSCN1 reversed EMT in many different cancer cells, as exhibited by corresponding changes in the expression of epithelial and mesenchymal markers.^79^^,^^97^^,^^101^^,^^111^ Furthermore, Mao et al.^79^ documented that FSCN1 promotes cholangiocarcinoma cell EMT by regulating Wnt/β-catenin signaling. FSCN1 can also directly interact with and elevate snail1 levels to promote EMT in ovarian cancer cells.^39^ In addition, several studies have shown that FSCN1 promotes tumor cell migration and invasion by upregulating the expression of multiple matrix metalloproteinases (MMPs)^101^^,^^116^^,^^126^^,^^179^ (Figure 5).

*Drug resistance*. Chemotherapeutic drug resistance is a growing concern in cancer management. Evidence suggests that FSCN1 expression in cancer cells is involved in chemoresistance (Tables 1 and 2). Ghebeh et al.^75^ revealed that FSCN1 regulates chemoresistance in breast cancer. The loss of FSCN1 expression sensitized breast cancer cells (MDA-MB-231) to doxorubicin therapy, whereas the expression of FSCN1 in the FSCN1-negative T47-D or SK-BR-3 breast cancer cell line conferred chemoresistance. Elevated chemoresistance levels in FSCN1-positive breast cancer cells are partially mediated through the phosphatidylinositol 3-kinase (PI3K)/Akt pathway.^75^ Moreover, FSCN1 expression in breast cancer cells confers resistance to chemotherapy by regulating the self-renewal abilities of breast cancer stem cells. Chemoresistance is a key feature of cancer stem cells. Indeed, Barnawi et al.^48^ reported that FSCN1 is involved in the chemotherapeutic resistance of breast cancer stem cells through the activation of the Notch self-renewal signaling pathway. They also showed that FSCN1-mediated expression of ITGB1 is important in several breast cancer cell functions, such as self-renewal and chemoresistance.^180^ In addition, FSCN1 expression in hepatocellular carcinoma cells^99^ and docetaxel-resistant lung adenocarcinoma cells^102^ was also found to enhance to chemoresistance. In these cells, FSCN1 regulates chemoresistance through the epithelial mesenchymal transition pathway.^99^^,^^102^ A recent study has revealed that FSCN1 enhances paclitaxel resistance in prostate cancer. Its expression abates miR-24-3p-mediated paclitaxel sensitivity in paclitaxel-resistant prostate cancer cells.^131^

*Cancer cell stemness*. In addition to the above-mentioned cell behaviors, upregulated FSCN1 also affects cancer cell stemness. Downregulated FSCN1 in breast cancer cell lines was shown to significantly suppress the cancer stem cell-like phenotype (CD44^hi^/CD24^lo^ and ALDH^+^).^48^ FSCN1 knockout in the melanoma WM793 cell line inhibited melanoma stemness. This was attributed to the fact that the expression levels of CD44 were significantly suppressed in FSCN1 knockout WM793 cells.^38^ Al-Alwan and coworkers^48^^,^^49^ documented that FSCN1 regulates breast cancer stem cell functions by activating the Notch and focal adhesion kinase (FAK)-β-catenin signaling pathway. Kang et al.^38^ revealed that FSCN1 increases cancer cell stemness in melanoma by inhibiting the Hippo pathway.

#### Is FSCN1 a cancer-driver gene?

Although FSCN1 promotes the progression of many human cancers, it is not currently listed as a cancer-driver gene. This is because the systematic sequencing of human cancer genomes has not revealed a significant rate of *FSCN1* somatic gene alterations.^181^ To our knowledge, a single study has evaluated the effects of *FSCN1* gene polymorphisms in the development and progression of breast cancer.^182^ The study documents that genetic variations in the *FSCN1* gene may serve as a marker for early-stage breast cancer. Even though FSCN1 is not directly affected by genetic mutations, it has an important role in regulating key oncogenic pathways, such as PI3K/AKT, Wnt/β-catenin, and MAPK. The available evidence is insufficient to assess the value of *FSCN1* as a new driver gene in several cancer cell lines. We therefore hypothesize that FSCN1 is a downstream effector protein that is upregulated to promote migration and invasion by remodeling cytoskeletal organization in response to various oncogenic signals in tumor cells.

In conclusion, FSCN1 is highly upregulated in a variety of human cancers and functionally contributes toward cancer progression. However, contrasting results have been reported in melanoma. Due to heterogeneity in different cancer cells and the complexity of multiple molecular mechanisms underlying tumor progression, evidence regarding FSCN1 roles in cancer development and progression is fragmented and limited. Therefore, integrated studies of these molecular mechanisms in the same cancer cells are needed to elucidate on the complex physiological functions of this gene.

### Is FSCN1 a promising cancer biomarker that can be used in clinical practice?

In spite of remarkable advancements in cancer research, it remains a major threat to human health. Effective cancer treatment depends on the implementation of novel therapeutic options, the development of methods for early-stage cancer diagnosis, and the assessment of an individual’s risk of cancer progression and recurrence. Cancer biomarkers in tumor tissues or serum can be used for risk evaluation, early diagnosis, tumor classification, prognosis, prediction of therapeutic responses or toxicity, and monitoring of cancer progression and recurrence.^183^ Therefore, effective cancer biomarkers are urgently needed to improve cancer screening, diagnosis, prognosis, and therapeutic monitoring. FSCN1 is a distinct 55-kDa actin-bundling protein that was identified as a sensitive marker for Reed-Sternberg cells of Hodgkin’s disease in 1997.^184^ The overexpression of FSCN1 in many other human cancers and their correlation with malignant properties have elucidated the important role of FSCN1 in cancer progression and prognosis. However, studies on the clinical significance of FSCN1 in human cancers are in the initial stages.

In the last 20 years, various clinical studies have documented that FSCN1 is a novel biomarker candidate for aggressive carcinomas of many cancer types.^1^^,^^3^ As shown in Table 3, we summarized the potential clinical use of FSCN1 as a biomarker in different human cancers and listed the level of evidence (LOE)^271^^,^^272^ for its clinical use. Clinical IHC studies showed that in nearly all cancers, FSCN1 expression is associated with aggressive clinical course, metastatic progression, and poor prognosis (Table 3). Cox regression analysis revealed that FSCN1 is an independent poor prognostic marker for many different human cancers (Table S2). FSCN1 can also serve as a diagnostic marker for distinguishing triple-negative subtypes from other breast cancer types,^71^^,^^203^^,^^209^ thyroid carcinoma from benign lesions,^267^ and uterine leiomyosarcoma from leiomyoma variants.^270^ Even though FSCN1 is not a secreted or membrane-bound protein, serum levels of FSCN1 play a role in cancer diagnosis and prognosis. Chen et al.^220^ revealed that FSCN1 autoantibodies are a potential serum diagnostic biomarker for ESCC, especially early-stage ESCC. Lee et al.^238^ showed that serum FSCN1 could distinguish between head and neck cancer patients from healthy individuals. Two retrospective studies have documented that serum FSCN1 levels are biomarkers for aggressive progression and prognosis of NSCLC.^245^^,^^246^ The clinicopathological and prognostic significance of tissue and serum FSCN1 levels in different human cancers is summarized in Table S2.

**Table 3:** The potential clinical applications of FSCN1 as a biomarker in human cancer

Cancer type	Specimen	Proposed use or comments	Level of evidence^a^	Methods	Refs.
Acute myeloid leukemia	plasma	distinguishing acute myeloid leukemia from acute lymphoblastic leukemia	III	ELISA	^185^
Adrenocortical cancer	tissue	independent poor prognostic marker; combined with stage or Ki67 LI, FSCN1 can refine their prognostication power	IV	IHC, WB, qRT-PCR	^186^^,^^187^
Ampulla of Vater adenocarcinomas	tissue	prediction of more malignant stage and poor survival	V	IHC	^188^
Biliary cancer	tissue	independent poor prognostic marker	IV	IHC, qRT-PCR	189, 190, 191, 192
	tissue	prediction of more aggressive clinical course and poor outcome	IV, V	IHC	^13^^,^^192^, ^193^, ^194^
Bladder cancer	tissue	independent prediction of recurrence and survival	IV	IHC	^195^^,^^196^
	tissue	prediction of invasion status in urothelial carcinomas	IV	IHC	197, 198, 199
	tissue	prediction of more malignant stage	IV, V	IHC	^70^^,^^200^
	tissue	an aid in the diagnosis of metastatic urothelial carcinoma	V	IHC	^201^
	tissue	does not correlate with the depth of tumor invasion or with the tumor recurrence or progression	III	IHC	^202^
Breast cancer	tissue	independent poor prognostic marker	IV	IHC	^71^^,^^203^, ^204^, ^205^, ^206^
	tissue	in combination with other factors for assessing prognosis in breast cancer	IV	IHC	^204^^,^^207^^,^^208^
	tissue	predicting prognosis after chemotherapy	V	IHC	^75^
	tissue	differential diagnosis of TNBC from other cancer types	IV	IHC	^71^^,^^203^^,^^209^
	tissue	prediction of more aggressive clinical course and poor outcome	IV, V	IHC	^12^^,^^67^^,^^76^^,^^206^^,^^210^
Childhood cancer	tissue	prediction of the risk of relapse	V	IHC	^211^
Colorectal cancer	tissue	independent poor prognostic marker for advanced colorectal cancer	IV	IHC	212, 213, 214, 215, 216, 217
	tissue	combination of FSCN1 and BMI1 as a prognostic marker	IV	IHC	^218^
	tissue	prediction of more aggressive clinical course and poor outcome	IV, V	IHC, qRT-PCR	^12^^,^^81^^,^^124^^,^^213^^,^^214^^,^^219^
	tissue	predicting distant metastasis	IV	IHC	^12^^,^^214^^,^^216^
Esophageal cancer	serum	early detection of ESCC	V	ELISA	^220^
	tissue	early detection of ESCC	IV	IHC	^221^^,^^222^
	tissue	independent poor prognostic marker	IV	IHC, qRT-PCR, WB	^87^^,^^160^^,^^223^, ^224^, ^225^, ^226^
	tissue	prediction of more aggressive clinical course and poor survival	IV	IHC, qRT-PCR	^12^^,^^222^^,^^224^^,^^227^
Gastric cancer	tissue	independent poor prognostic marker	IV	IHC	^228^^,^^229^
	tissue	combined with Smad4 for predicting clinical outcomes	IV	IHC	^230^
	tissue	predicting lymph node or distant metastasis	IV	IHC	^12^^,^^229^^,^^231^
	tissue	prediction of more aggressive clinical course and poor outcome	IV	IHC	^12^^,^^229^^,^^231^, ^232^, ^233^, ^234^
Gastrointestinal stromal tumor	tissue	prediction of aggressive behavior and poor outcome	IV	IHC	^163^
Glial tumor	tissue	independent poor prognostic marker	IV, V	IHC, qRT-PCR	235, 236, 237
Head and neck cancer	serum	differentiating cancer patients from healthy individuals	IV	ELISA	^238^
	tissue	prediction of regional lymphatic metastasis	III, IV	IHC	^238^^,^^239^
Hepatocellular carcinoma	tissue	prediction of more aggressive clinical course and poor outcome	IV, V	IHC	^240^^,^^241^
	tissue	independent prognostic factor for disease-free survival	IV	IHC	^241^
Laryngeal cancer	tissue	independent poor prognostic marker	IV	IHC	^101^^,^^242^^,^^243^
	tissue	combined with E-cadherin for predicting recurrence	V	IHC	^243^
	tissue	prediction of more aggressive clinical course	IV, V	IHC	^101^^,^^242^^,^^244^
Lung cancer	serum	predicting recurrence in NSCLC	V	ELISA	^245^
	serum	independent prognostic marker for M0-stage NSCLC; prediction of metastasis	IV	ELISA	^246^
	tissue	prediction of more aggressive clinical course	IV, V	IHC	^4^^,^^91^^,^^127^^,^^247^, ^248^, ^249^, ^250^
	tissue	independent poor prognostic marker for NSCLC patients	IV	IHC, qRT-PCR, WB	^4^^,^^91^^,^^249^
Nasopharyngeal cancer	tissue	independent poor prognostic marker; prediction of more malignant status	IV	IHC	^105^
Oral cancer	tissue	independent poor prognostic marker	IV, V	IHC	^111^^,^^251^^,^^252^
	tissue	prediction of more aggressive clinical course and poor outcome	IV, V	IHC	^121^^,^^128^^,^^251^, ^252^, ^253^
Osteosarcoma	tissue	prediction of poor overall survival	V	IHC	^129^
Ovarian cancer	tissue	independent poor prognostic marker for survival of advanced serous ovarian cancer	IV, V	IHC	^114^^,^^254^
	tissue	prediction of more aggressive clinical course and poor survival	IV, V	IHC	^114^^,^^255^, ^256^, ^257^, ^258^
Pancreatic cancer	tissue	prediction of more malignant stage and poor survival	V	IHC	^152^^,^^188^^,^^259^^,^^260^
Pituitary adenomas	tissue	prediction of invasion and risk of recurrence	V	IHC	^261^
Prostate cancer	tissue	prediction of more aggressive clinical course	V	IHC	^10^
	tissue	not a suitable biomarker for prediction of aggressive prostate cancers	IV	IHC	^262^
Renal cancer	tissue	independent poor prognostic marker	IV, V	IHC	^120^^,^^133^
	tissue	prediction of more malignant stage and poor survival	IV, V	IHC	^263^^,^^264^
Small intestinal carcinoma	tissue	independent poor prognostic marker; prediction of lymph node metastasis	IV	IHC	^265^
Soft tissue sarcomas	tissue	prediction of disease-specific survival	V	IHC	^266^
Thyroid cancer	tissue	distinguishing thyroid carcinoma from benign lesions	IV	IHC	^267^
Uterine cancer	tissue	prediction of more aggressive clinical course and poor outcome	V	IHC	^268^^,^^269^
	tissue	differentiating uterine leiomyosarcoma from leiomyoma variants	V	IHC	^270^

IHC, immunohistochemistry of conventional tissue sections; ELISA, enzyme-linked immunosorbent assay; qRT-PCR, quantitative reverse transcriptase polymerase chain reaction; WB, western blot; TNBC, triple-negative breast cancer.

a Level of evidence:^271^ level I, evidence from a single, high-powered, prospective, controlled study that is specifically designed to test the marker or evidence from a meta-analysis, pooled analysis, or overview of level II or III studies; level II, evidence from a study in which marker data are determined in relationship to a prospective therapeutic trial that is performed to test a therapeutic hypothesis but not specifically designed to test marker utility; level III, evidence from large prospective studies; level IV, evidence from small retrospective studies; level V, evidence from small pilot studies.

The LOE reflects the strength of current evidence for clinical utility of a biomarker. According to the criteria for LOE, as defined by Hayes et al.,^271^ LOE I represents the highest evidence, whereas LOE V represents the poorest evidence for the clinical utility of a particular biomarker. Although FSCN1 is a promising biomarker candidate for aggressive carcinomas, its current LOE is low (level IV or V; Table 3). To our knowledge, only three prospective studies have a higher LOE (level III).^202^^,^^219^^,^^239^ There are several explanations for this low LOE. Many studies were piloted to determine FSCN1 expression in sample populations or to correlate FSCN1 levels with clinicopathological parameters. These studies were not designed to determine the clinical utility of FSCN1; therefore, their LOE was level V. Moreover, most of these studies were designed retrospectively, thereby providing a low LOE (level IV) when compared to prospectively designed studies (level III). Several systematic reviews and meta-analyses have established that FSCN1 is a potential biomarker for the identification of aggressive metastatic tumors or prognosis.^5^^,^^12^^,^^13^^,^^189^^,^^223^^,^^232^ However, the evidence from these retrospectively reviews cannot be considered as higher LOE. To improve the LOE, studies should preferably select appropriate samples from previously established prospective cohorts.

Since the current LOE for clinical utility of FSCN1 is low, it raises an important question: how far are we from using FSCN1 as a cancer biomarker in clinical practice? Several studies have discussed the process of biomarker development (e.g., Pepe et al.,^273^ Rifai et al.,^274^ and Pavlou et al.^275^). A simplistic version of a biomarker development pipeline^275^ includes 4 sequential phases: phase 1, preclinical exploratory studies; phase 2, clinical assay development; phase 3, retrospective validation studies; and phase 4, prospective validation studies. Various challenges at different levels must be overcome before a biomarker moves from one phase to the other. Only biomarkers that successfully reach phase 4 are approved for clinical use.^273^, ^274^, ^275^

The expression of FSCN1 is associated with an aggressive clinical course and poor outcomes in different cancer types (Tables 3 and S2). Therefore, FSCN1 seems a promising cancer biomarker. However, in (nearly) all cancer types, FSCN1 does not progress beyond phase 2 of the biomarker development pipeline.^275^ This has been attributed to the lack of retrospective validation studies. In contrast to the retrospective studies in phase 1 or 2, retrospective validation studies (phase 3) require bigger sample sizes that reflect the biological variability of the targeted population to ensure a rigorous statistical analysis. The vast majority of published studies was retrospectively designed to investigate the associations between FSCN1 levels with clinicopathological parameters or outcomes of interest. The aim of these studies was to explore or evaluate the potential of FSCN1 as a biomarker (e.g., the studies in gastric^228^^,^^229^ and liver^240^^,^^241^ cancer). In addition, FSCN1, as an independent poor prognostic marker for aggressiveness, has been evaluated in multiple patient populations of some cancers (e.g., breast, colon, and esophageal cancer). However, the available evidence is insufficient for assessing the independent value of fascin-1 as a new biomarker. This is because individual studies are not always consistent.^12^ Furthermore, only a few relevant studies have been performed to investigate the potential of FSCN1 as a biomarker in certain types of human cancer (Table 3). Apart from breast cancer,^12^ inconsistent results were also found in several other cancer types, such as bladder^195^^,^^196^^,^^202^ and prostate^10^^,^^262^ cancer. Therefore, prospective studies with bigger sample sizes are also needed to fully determine the predictive or prognostic value of FSCN1.

Several reasons have been postulated as to why FSCN1 has not been approved as a biomarker in clinical practice.^3^^,^^5^^,^^6^^,^^12^ First, there is a lack of sufficient evidence to assess the independent value of FSCN1 as a new biomarker.^5^^,^^12^ Contrasting findings between individual studies are not conclusive as to whether FSCN1 is a reliable biomarker, therefore. Contrasting results inhibit large-scale and well-designed prospective studies. Second, because FSCN1 is not a secreted or membrane-bound protein, it cannot be used as a serum biomarker for different cancer types. Third, previous studies have shown that FSCN1 is highly expressed in many different cancer types (Table S1); therefore, it is not specific to any tumor tissue type to be a good biomarker. More studies should be done to determine the clinical relevance and applicability of FSCN1 in the most relevant cancers.

### Therapeutic potential of FSCN1

Evidence shows that FSCN1 is a viable novel target molecule for anti-cancer or anti-metastatic therapy in multiple human cancers.^1^^,^^5^^,^^7^^,^^10^^,^^12^^,^^121^ As a therapeutic target, FSCN1 has several advantages: (1) it is important for tumor progression and promotes tumor cell migration, invasion, and metastasis (refer to Tables 1 and 2); (2) it is upregulated in many human cancers and has been correlated with clinically aggressive phenotypes and poor prognosis (refer to Table 3); and (3) FSCN1 knockout mice are viable,^61^^,^^276^ suggesting that targeting FSCN1 would have limited side effects in patients. Currently, siRNA, shRNA, miRNAs, small molecule inhibitors, and nanobodies have been experimentally used for targeting FSCN1.

siRNA can be used for specific degradation of targeted mRNA and therefore, reducing protein abundance. This enhances the utilization of siRNA as a powerful tool to study the functions of a specific gene. In most of the tumor cell lines (Table 1), FSCN1 knockdown by siRNA inhibited cell migration and invasion *in vitro*. Several studies of multiple cancer types have also shown that the downregulation of FSCN1 through siRNA or shRNA is effective at suppressing tumor cell metastasis *in vivo*.^10^^,^^55^^,^^65^^,^^95^^,^^96^^,^^115^^,^^120^ These studies suggest that using siRNA to downregulate FSCN1 expression in tumors may be a new class of therapeutics for metastatic cancers. However, the clinical feasibility of using siRNA as a therapeutic option is hampered by its off-target effects.

In addition, studies performed in multiple cancer cell lines indicate that overexpression of miRNAs, such as miR-145, inhibits cell growth, cell migration, and invasion by directly targeting FSCN1.^82^^,^^101^^,^^117^ However, the clinical applications of miRNAs or miRNA-based reagents as therapeutic options have significant limitations.^277^

Small molecule inhibitors of FSCN1 can block tumor cell migration, invasion, and metastasis.^7^^,^^8^^,^^14^^,^^15^^,^^278^ Moreover, this category of inhibitors could potentially be useful against FSCN1-positive tumors from different tissues. In the last 10 years, FSCN1 has been identified as a protein target for a number of small molecule compounds. In 2010, Huang and coworkers^7^ showed that migrastatin analogs can block tumor metastasis by targeting FSCN1 to inhibit its activity. Small molecule compound G2 and its improved analogs have also been shown to inhibit the actin-binding activity of FSCN1, tumor cell migration and invasion, and metastasis in mouse models.^8^^,^^14^^,^^15^ Alburquerque-González et al.^278^ demonstrated that the anti-depressant imipramine inhibits FSCN1 and plays a role in inhibiting the migration and invasion of colorectal cancer cells. A series of thiazole derivatives,^32^^,^^33^ isoquinolone and pyrazolo[4,3-c] pyridine,^34^ have also been shown to be inhibitors of FSCN1. However, these small molecule compounds provide promising start points for the development of FSCN1-targeted anti-metastatic therapies.

Another approach that targets the FSCN1 protein is the use of an inhibitory nanobody that disrupts FSCN1/actin bundling.^279^ When expressed inside prostate cancer cells (PC3) or breast cancer cells (MDA-MB-231), FSCN1-specific nanobodies inhibited invadopodium formation and cell invasion.^279^ However, it has not been established whether this FSCN1-specific nanobody can be developed for clinical applications.

The possible utilization of FSCN1 as a therapeutic target is inhibited by multiple limitations. Studies are aimed at developing novel agents that can interfere with key FSCN1 functions in cancers.^8^^,^^33^^,^^278^, ^279^, ^280^ It is foreseeable that creative agents (siRNAs, small molecule inhibitors, nanobody, etc.) will be developed to specifically inhibit FSCN1-mediated tumor metastasis. However, FSCN1 expression can promote cell migration and metastasis independent of its actin-bundling activity.^36^^,^^44^^,^^45^^,^^63^ This should be taken into consideration when developing therapeutic options that inhibit FSCN1/actin-bundling activity.

### Conclusions

Studies have shown that FSCN1 is expressed in many human cancer types. Its expression has been correlated with aggressive clinical course and poor prognosis.^1^^,^^9^^,^^11^^,^^12^^,^^281^ *In vitro* manipulation of FSCN1 expression in tumor cell lines has shown that FSCN1 promotes tumor cell growth, migration, invasion, and metastasis (Tables 1 and 2). Furthermore, FSCN1 is involved in the regulation of key oncogenic pathways, such as EMT, PI3K/AKT, Wnt/β-catenin, MAPK, among others (Figure 5). Therefore, FSCN1 is a potential biomarker for aggressive, metastatic cancers^6^^,^^9^^,^^12^ and is a therapeutic target for blocking tumor cell migration, invasion, and metastasis.^3^^,^^7^^,^^8^^,^^10^^,^^14^^,^^282^ However, it has not been established whether FSCN1 can be developed as a novel biomarker or therapeutic target. More studies are needed to determine whether FSCN1 has value as a biomarker in the most relevant cancers, over biomarkers that are in current clinical use, and whether targeting FSCN1 with small molecules will be useful for cancer therapy.

In addition, due to heterogeneity in different cancer cells and the complexity of multiple molecular mechanisms underlying tumor progression, evidence regarding FSCN1 roles in cancer development and progression is fragmented and limited. Therefore, much remains to be learned about the role of FSCN1 in human cancers. For example, FSCN1 is important for cancer cell stemness,^38^^,^^48^^,^^49^ extracellular vesicle release,^47^ chemoresistance,^75^ and anoikis resistance,^283^ yet the mechanisms involved are largely unclear. Investigation of the relationship among mitochondrial metabolism, FSCN1, and metastatic colonization in cancers has begun;^65^ more widespread investigations can be expected in the next few years. Although FSCN1 overexpression has been extensively reported in different human cancers, molecular mechanisms underlying FSCN1 upregulation during malignant transformation and metastatic progression are under-studied areas. It is also unknown whether super enhancers, N6-methyladenosine (m6A) modification, or RNA binding proteins play a role in the regulation of FSCN1 expression. Understanding these roles and mechanistic regulation of FSCN1 in cancers will be crucial for the development of therapeutic interventions targeting FSCN1.

## Supplemental information

Table S1. FSCN1 expression in human cancer and normal epithelium/para-carcinomas tissues

Table S2. Clinicopathological and prognostic significance of tissue and serum FSCN1 levels in different human cancers
