# Supplementary material for: Fascin actin-bundling protein 1 in human cancer: promising biomarker or therapeutic target?
Source: Mol Ther Oncolytics. 2021 Jan 20;20:240–64. doi: 10.1016/j.omto.2020.12.014 (PMC7873579; doi:10.1016/j.omto.2020.12.014)
Supplement: Table S1. FSCN1 expression in human cancer and normal epithelium/para-carcinomas tissues [file mmc1.pdf]

**OMTO, Volume *20***

## Supplemental Information

# Fascin actin-bundling protein 1 in human cancer: promising biomarker or therapeutic target?

**Hongliang Liu, Yu Zhang, Li Li, Jimin Cao, Yujia Guo, Yongyan Wu, and Wei Gao**

**Table S1:** FSCN1 expression in human cancer and normal epithelium/para-carcinomas tissues.

| Tissue/organ (cancer type) | Normal epithelium or para-carcinoma | Cancer tissues | Methods | Reference |
|---|---|---|---|---|
| Adrenal glands (adrenocortical carcinoma) | Not detected | High expression in 79% (26/33, IHC); High expression in 71% (15/21, WB); | IHC, WB | 1 |
| Ampulla (ampulla of Vater adenocarcinomas) | Not detected | Positive expression in 70% (28/40) | IHC | 2 |
| Ampulla (ampulla of Vater adenocarcinomas) | Not detected | Positive expression in 77% (42/54) | TMA | 3 |
| Biliary duct (gallbladder cancer) | Not done | High expression in 55.8% (24/43) | IHC | 4 |
| Biliary duct (gallbladder adenocarcinomas) | Absent | Positive expression in 58.3% (28/48) | TMA | 5 |
| Biliary duct (intrahepatic cholangiocarcinoma) | Absent or sporadic expression | High expression in 35.7% (30/84) | IHC | 6 |
| Biliary duct (cholangiocarcinoma) | Not detected (0/20) | Positive expression in 63% (89/142) | IHC | 7 |
| Biliary duct (cholangiocarcinoma) | Not done | Positive expression in 49% (43/87) | IHC | 8 |
| Biliary duct (extrahepatic bile duct carcinomas) | Not done | Positive expression in 57% (65/114) | IHC | 9 |
| Bladder (bladder cancer) | Not detected | Overexpression in 83.2% (104/125) | IHC | 10 |
| Bladder (metastatic urothelial carcinomas) | Not done | Overexpression in 92% (23/25), and positive staining in 100% | IHC | 11 |
| Bladder (urothelial carcinoma) | Not detected (0/42) | Positive expression in 84.7% (94/111) | IHC | 12 |
| Bladder (urothelial carcinoma) | Not detected | Diffuse strong staining in 95% (19/20) of invasive carcinoma | IHC | 13 |
| Bladder (urothelial carcinomas) | Not done | Strong staining in 61.1% (77/126) of invasive carcinomas | IHC | 14 |
| Bladder (urothelial carcinomas) | Not detected (0/32) | Positive expression in 84.1% (74/88) | IHC | 15 |
| Bladder (urothelial carcinomas) | Positive expression in 20% (5/25) | Positive expression in 63% (77/122) | TMA | 16 |
| Brain (primary glioblastoma) | Not done | Low expression in 35.1% (13/37); high expression in 64.9% (24/37) | IHC | 17 |
| Brain (glioma) | Low expression (34/120) | High expression in 46.7% (56/120) | IHC, RT-PCR | 18 |
| Brain (various cancer types) | Positive in normal neurons | High expression in 50% (22/44) of glioblastomas | IHC | 19 |

| | | | | |
|---|---|---|---|---|
| Brain (skull base chordoma) | Not done | High expression in 66.7% (26/39) | IHC | 20 |
| Breast (breast carcinomas) | Negative | Positive expression in 16% (33/210) | IHC | 21 |
| Breast (Various tumor types) | Not done | Positive expression in 6.4% (8/125) of usual ductal hyperplasia, 17.3% (18/104) of ductal carcinoma in situ and 33.0% (154/467) of invasive ductal carcinoma | IHC | 22 |
| Breast (breast cancer) | Not done | Positive expression in 20.1% (48/239) | IHC | 23 |
| Breast (breast carcinoma) | Positive in myoepithelial cells and luminal cells of few ducts and acini | Positive expression in 43.3% (29/67) | IHC | 24 |
| Breast (infiltrating duct carcinoma) | Not done | Positive expression in 51.4% (72/140) | IHC | 25 |
| Breast (invasive ductal carcinoma) | Not done | Positive expression in 21.1% (41/194) | TMA | 26 |
| Breast (node-negative breast cancer) | Not done | Positive expression in 18% (33/183) | IHC | 27 |
| Breast (node-negative invasive breast carcinomas) | weakly expression | Positive expression in 25.1% (56/224) of sporadic invasive breast carcinomas | TMA | 28 |
| Breast (primary breast carcinomas) | Not done | Positive expression in 40.6% (82/202) | TMA | 29 |
| Breast (triple-negative breast cancer) | Not done | Positive expression in 31.5% (144/457) | IHC | 30 |
| Cervix (cervical neoplasms) | Not done | Positive expression in 94% (31/33) of squamous intraepithelial lesions and 67% (31/46) of invasive cervical carcinoma | IHC | 31 |
| Cervix (endocervical neoplasia) | Negative | Positive expression in 8% (2/25) of ACIS lesions and 40.6% (13/32) of adenocarcinomas | IHC | 32 |
| Cervix (superficially invasive (stage IA1) squamous carcinoma) | Positive in basal and parabasal cells | Positive expression in 66.7% (18/27) | IHC | 33 |
| Colon (colorectal adenocarcinomas) | Not detected | Positive expression in 16% (17/107) of adenomas and 26% (9/35) of adenocarcinomas | TMA | 34 |
| Colon (colorectal adenocarcinoma) | Scattered expression in 9 normal epithelia | Higher immunostaining scores in 91 carcinomas | TMA | 35 |
| Colon (colorectal adenomas) | Not detected (0/10) | Positive expression in 73.4% (47/64) | IHC | 36 |

| | | | | |
|---|---|---|---|---|
| Colon (colorectal cancer) | Positive expression in 10.4% (45/433) of normal tissues | Weakly expression in 10.3% (46/446), intermediate in 18.8% (84/446) and strongly in 12.3% (55/446) | TMA | 37 |
| Colon (stage III colorectal cancer) | Not detected | Positive expression in 58.7% (74/126) | IHC | 38 |
| Colon (stage III-IV colonic adenocarcinoma) | Not detected | Positive expression in 71% (162/228) | IHC | 39 |
| Colon (colorectal adenocarcinoma) | Not done | High expression in 24% (50/210) | IHC | 40 |
| Colon (colorectal cancer) | Not detected | Low expression in 25.5% (13/51) and high expression in 74.5% (38/51) | IHC | 41 |
| Colon (colorectal carcinomas) | Not detected | Positive expression in 35.3% (59/167) | IHC | 42 |
| Ear (cholesteatoma) | No (7/28) or very low expression (21/28) | All positive, high expression in 71.4% (20/28) of cholesteatoma | IHC | 43 |
| Endometrium (endometrial carcinomas) | Not done | Positive expression in 81.9% (9/11) | IHC | 44 |
| Endometrium (endometrioid carcinoma) | Weakly expression in 30% (3/10) | Positive expression in 72.34% (34/47) | IHC | 45 |
| Endometrium (endometrioid carcinoma) | Weakly expression in 39% of glandular epithelium | High expression in 74% (52/71) of neoplastic | IHC | 46 |
| Endometrium (endometrial carcinoma) | Not done | Positive expression in 91% (20/22) | IHC | 47 |
| Esophagus (esophageal squamous cell carcinoma) | Not done | Positive expression in 58.6% (82/140) | IHC,qPCR | 48 |
| Esophagus (esophageal squamous cell carcinoma) | Positive expression in 26% (9/35) | Positive expression in 56% (129/231) | TMA | 49 |
| Esophagus (esophageal squamous cell carcinoma) | Not done | Positive expression in 35.4% (90/254) | IHC | 50 |
| Esophagus (esophageal squamous cell carcinoma) | Positive in basal layer | High expression of fascin detected in 87.76% (43/49) using WB and 77.55% (38/49) using RT-PCR | IHC, WB, qPCR | 51 |
| Esophagus (esophageal squamous cell carcinoma) | Positive in basal and lower spinous layers | Positive expression in 71.1% (145/200) | IHC | 52 |
| Gastrointestinal tract (gastrointestinal stromal tumor) | Not done | Positive expression in 47.6% (70/147) | IHC | 53 |
| Gastrointestinal tract (gastrointestinal stromal tumor) | Not done | Positive expression in 16.7% (5/30) | IHC | 54 |

| | | | | |
|---|---|---|---|---|
| Head and neck (nasopharyngeal carcinoma) | Detected in 18.2% (6/33) | High expression in 59.0% (95/161) | IHC | 55 |
| Head and neck（squamous cell carcinoma ） | Moderate positive in 40% (8/20) | Moderate positive in 52.2% (12/23), strong positive in 43.4% (10/23) | IHC | 56 |
| Head and neck (oral squamous cell carcinoma) | Not done | Low expression in 55% (22/40) and high expression in 45% (18/40) | IHC | 57 |
| Head and neck (laryngeal squamous cell carcinoma) | Low or absent | Positive expression in 91.7% (198/216) | qPCR, WB, IHC | 58 |
| Head and neck (laryngeal squamous cell carcinoma) | Not done | Positive expression in 83.3% (25/30) | IHC | 59 |
| Head and neck (laryngeal squamous cell carcinoma) | Not done | High expression in 52.7% (79/150) | IHC | 60 |
| Head and neck (oral and oropharyngeal squamous cell carcinomas) | Weakly positive staining | Weakly, moderate and intensely positive staining in 30.2% (39/129), 58.9% (76/129) and 10.9% (14/129), respectively | IHC | 61 |
| Head and neck (oral squamous cell carcinoma) | Weak to no expression | Positive expression in 91.3% (42/46) | IHC | 62 |
| Head and neck (oral squamous cell carcinoma) | Weak or negative in 5 oral mucosae | Intensely positive staining in 32.28% (43/131), weakly in 41.98% (55/131) | IHC | 63 |
| Head and neck (tongue squamous cell carcinoma) | Low expression | High expression in 62.2% (69/106) | IHC | 64 |
| Intestine (small intestinal carcinoma) | Not done | Positive expression in 24.2% (47/194) | TMA | 65 |
| Kidney (non-metastatic clear-cell renal cell carcinoma) | Low expression | Low expression in 56.2% (109/194) and high expression in 43.8% (85/194) | IHC | 66 |
| Kidney (renal cell carcinoma) | Positive expression in 26% (6/23) | Positive expression in 71.1% (37/52) | IHC | 67 |
| Kidney (renal cell carcinoma) | Not detected | Positive expression in 100 various subtypes of renal cell carcinoma | TMA | 68 |
| Kidney (renal cell carcinoma) | Not done | Positive expression in 10% (13/136) of primary and 46% (25/54) of metastatic renal cell carcinoma | TMA | 69 |
| Kidney (renal cell carcinoma) | Not detected | High expression in 108 renal cell carcinoma specimens | IHC | 70 |
| Liver (hepatocellular Carcinoma) | Absent or sporadic in normal epithelium | Positive expression in 16.8% (23/137) | IHC | 71 |
| Liver (hepatocellular carcinoma) | Done but not described | High expression in 10 carcinoma Tissues | qPCR，WB | 72 |
| Liver (hepatocellular carcinoma) | Positive expression in 14.2% (1/7) | Positive expression in 61% (47/77) | IHC | 73 |

| | | | | |
|---|---|---|---|---|
| Liver (hepatocellular carcinoma) | Not done | Positive expression in 19% 10/53) | IHC | 74 |
| Lung (lung cancer) | Positive expression in 32.1% (27/84) of para-carcinoma | Positive expression in 92.8% (78/84) | IHC | 75 |
| Lung (non-small cell lung cancer) | Positive expression in 28.4% (23/81) of para-carcinoma | Weakly expression in 35.8% (29/81) and strongly expression in 40.7% (33/81) | IHC | 76 |
| Lung (non-small cell lung cancer) | Not done | Positive expression in 21.1% (80/378) | IHC | 77 |
| Lung (non-small cell lung cancer) | Positive expression in 13.1% (8/61) | Positive expression 70.5% (43/61) | IHC | 78 |
| Lung (non-small cell lung cancer) | Weak to no expression in 31 para-carcinoma | High expression in 31 carcinoma tissues | qPCR, WB | 79 |
| Lung (non-small cell lung cancer) | High expression in 26.9% (7/26) of normal tissues | High expression in 53.2% (83/156) of carcinomas | IHC | 80 |
| Lung (non-small cell lung cancer) | High expression in 27.3% (35/128) of adjacent tissues | High expression in 57% (73/128) of carcinomas | IHC | 81 |
| Lung (pulmonary adenocarcinomas) | Not detected | Positive expression in 61.2% (30/49) | TMA | 82 |
| Lung (pulmonary carcinoids) | Negative | Positive expression in 5% (2/38) of typical carcinoids, 35% (8/23) of atypical carcinoids, 83% (33/40) of large-cell neuroendocrine carcinomas and 100% (27/27) of small-cell lung carcinomas | IHC | 83 |
| Lung (various lung cancer types) | Not done | Positive expression in 98% (113/116) of squamous cell carcinomas, 78% (75/96) of adenocarcinomas, 83% (5/6) large cell carcinomas and 2 adenosquamous carcinomas | IHC | 84 |
| Ovary (primary mucinous ovarian tumors) | Negative | Positive staining in 84% (68/81) | TMA | 85 |
| Ovary (Various cancer types) | Negative (0/10) | Positive expression in 65% (13/20) of borderline and 84% (22/26) of malignant serous tumors | IHC | 86 |
| Ovary (Various cancer types) | Very low or absent | High expression in 172 ovarian tumors | TMA | 87 |
| Ovary (advanced poorly differentiated serous ovarian cancer) | Not done | High expression in 37.5% (21/56) and low expression in 62.5% (35/56) | IHC | 88 |

| | | | | |
|---|---|---|---|---|
| Ovary (borderline ovarian tumors) | Not done | Positive expression in 51.4% (72/140) | IHC | 89 |
| Ovary (epithelial ovarian cancer) | Not detected | Weakly expression in 33.7% (30/89) and strongly expression in 60.7% (54/89) | IHC | 90 |
| Ovary (four ovarian cancer types) | Not done | Higher positive staining in 185 tumors | TMA | 91 |
| Ovary (high-grade serous ovarian carcinoma) | Not done | Positive expression in 45.5% (36/79) | TMA | 92 |
| Pancreas (pancreatic adenocarcinomas) | Not detected | Positive expression in 70% (35/50) | IHC | 2 |
| Pancreas (intraductal papillary mucinous neoplasms) | Not done | High expression in 86% (25/29) of borderline neoplasms and 88% (37/42) of carcinomas | IHC | 93 |
| Pancreas (pancreatobiliary carcinoma) | Not detected | Higher positive staining in 100 tumours | TMA | 94 |
| Pituitary (pituitary adenomas) | Not done | High expression in 43.9% (137/312) | IHC | 95 |
| Prostate (prostate cancer) | Detected in 29% (57/196) of benign prostate tissues | Increased expression in 70% (137/196) of prostate cancer | TMA | 96 |
| Skin (melanomas) | Not done | High expression in 10.2 % (26/254) of primary melanomas and 35.4 % (17/48) of melanoma metastasis | IHC | 97 |
| Skin (various cancer types) | Positive in normal skin spinous and basal keratinocytes, melanocytes, and endothelial cells | Positive expression in 73 % (19/26) of squamous cell carcinoma, 100% (9/9) basal cell carcinoma, 19% (3/16) of melanoma, and 60% (3/5) of merkel cell carcinoma | IHC | 98 |
| Soft tissue (non-gastrointestinal stromal tumor soft tissue sarcomas) | Not done | High expression in 36.5% (91/249) and low expression in 61.4% (153/249) | TMA | 99 |
| Stomach (gastric adenocarcinoma) | Not done | Positive expression in 47% (47/100) | IHC | 100 |
| Stomach (gastric cancer) | Positive expression in 27.5% of adjacent cancer tissues | Positive expression in 45.1% (92/204) | IHC，WB | 101 |
| Stomach (gastric cancer) | Not done | Positive expression in 14.9% (70/471) | TMA | 102 |
| Stomach (gastric carcinoma) | Not done | Positive expression in 25.2% (54/214) | IHC | 103 |
| Thyroid (thyroid neoplasms) | Negative | Positive expression in 62.1% (41/66) of thyroid carcinomas and 26.4% (19/72) of thyroid adenomas | IHC | 104 |

| | | | | |
|---|---|---|---|---|
| Uterus (Various smooth muscle tumours) | Not done | Positive expression in 90.9% (20/22) of leiomyosarcoma | IHC | 105 |
| Uterus (uterine carcinosarcoma) | Negative | High expression in 47.7% (21/44) | IHC | 106 |

**IHC: immunohistochemistry; TMA: tissue microarray; WB: western blot; qPCR: Real-time Quantitative-Polymerase Chain Reaction**

## REFERENCES


1. Poli, G., Ruggiero, C., Cantini, G., Canu, L., Baroni, G., Armignacco, R., Jouinot, A., Santi, R., Ercolino, T., Ragazzon, B., et al. (2019). Fascin-1 Is a Novel Prognostic Biomarker Associated With Tumor Invasiveness in Adrenocortical Carcinoma. J. Clin. Endocrinol. Metab.. *104*, 1712-1724.
2. Tsai, W.C., Lin, C.K., Lee, H.S., Gao, H.W., Nieh, S., Chan, D.C., and Jin, J.S. (2013). The correlation of cortactin and fascin-1 expression with clinicopathological parameters in pancreatic and ampulla of Vater adenocarcinoma. APMIS. *121*, 171-181.
3. Van Heek, N.T., Maitra, A., Koopmann, J., Fedarko, N., Jain, A., Rahman, A., Iacobuzio-Donahue, C.A., Adsay, V., Ashfaq, R., Yeo, C.J., et al. (2004). Gene expression profiling identifies markers of ampullary adenocarcinoma. Cancer Biol. Ther.. *3*, 651-656.
4. Roh, Y.H., Kim, Y.H., Choi, H.J., Lee, K.E., and Roh, M.S. (2009). Fascin overexpression correlates with positive thrombospondin-1 and syndecan-1 expressions and a more aggressive clinical course in patients with gallbladder cancer. J Hepatobiliary Pancreat Surg. *16*, 315-321.
5. Swierczynski, S.L., Maitra, A., Abraham, S.C., Iacobuzio-Donahue, C.A., Ashfaq, R., Cameron, J.L., Schulick, R.D., Yeo, C.J., Rahman, A., Hinkle, D.A., et al. (2004). Analysis of novel tumor markers in pancreatic and biliary carcinomas using tissue microarrays. Hum. Pathol.. *35*, 357-366.
6. Iguchi, T., Aishima, S., Taketomi, A., Nishihara, Y., Fujita, N., Sanefuji, K., Sugimachi, K., Yamashita, Y., Maehara, Y., and Tsuneyoshi, M. (2009). Fascin overexpression is involved in carcinogenesis and prognosis of human intrahepatic cholangiocarcinoma: immunohistochemical and molecular analysis. Hum. Pathol.. *40*, 174-180.
7. Mao, X., Chen, D., Wu, J., Li, J., Zhou, H., Wu, Y., and Duan, X. (2013). Differential expression of fascin, E-cadherin and vimentin: Proteins associated with survival of cholangiocarcinoma patients. Am. J. Med. Sci.. *346*, 261-268.
8. Onodera, M., Zen, Y., Harada, K., Sato, Y., Ikeda, H., Itatsu, K., Sato, H., Ohta, T., Asaka, M., and Nakanuma, Y. (2009). Fascin is involved in tumor necrosis factor-alpha-dependent production of MMP9 in cholangiocarcinoma. Lab. Invest.. *89*, 1261-1274.
9. Won, K.Y., Kim, G.Y., Lim, S.J., Park, Y.K., and Kim, Y.W. (2009). Prognostic significance of fascin expression in extrahepatic bile duct carcinomas. Pathol. Res. Pract.. *205*, 742-748.
10. El-Rehim, D.M., El-Maqsoud, N.M., El-Hamid, A.M., El-Bab, T.K., and Galal, E.M. (2013). Expression of extracellular matrix metalloproteinase inducer and fascin in urinary bladder cancer: Correlation with clinicopathological characteristics. Mol Clin Oncol. *1*, 297-304.
11. Vogt, A.P., Cohen, C., and Siddiqui, M.T. (2012). Fascin as an identifier of metastatic urothelial carcinoma: A retrospective study of fine-needle aspiration cell blocks and histologic tissue microarrays. Diagn. Cytopathol.. *40*, 882-886.
12. Bi, J., Chen, X., Zhang, Y., Li, B., Sun, J., Shen, H., and Kong, C. (2012). Fascin is a predictor for invasiveness and recurrence of urothelial carcinoma of bladder. Urol. Oncol.. *30*, 688-694.
13. Tong, G.X., Yee, H., Chiriboga, L., Hernandez, O., and Waisman, J. (2005). Fascin-1 expression in papillary

and invasive urothelial carcinomas of the urinary bladder. Hum. Pathol.. *36*, 741-746.

14. Sharma, A., Badwal, S., Dutta, V., and Basu, A. (2014). Evaluation of fascin-1 expression as a marker of invasion in urothelial carcinomas. Med J Armed Forces India. *70*, 139-143.
15. Bi, J.B., Zhu, Y., Chen, X.L., Yu, M., Zhang, Y.X., Li, B.X., Sun, J.W., Shen, H.L., and Kong, C.Z. (2013). The role of fascin in migration and invasion of urothelial carcinoma of the bladder. Urol. Int.. *91*, 227-235.
16. Gomaa, W., Al-Maghrabi, H., Al-Attas, M., Al-Ghamdi, F., and Al-Maghrabi, J. (2019). Fascin expression in urinary bladder urothelial carcinoma correlates with unfavourable prognosis. Int J Clin Exp Pathol. *12*, 3901-3907.
17. Park, K.S., Lee, H.W., Park, S.H., Park, T.I., and Hwang, J.H. (2016). The clinical significance of fascin expression in a newly diagnosed primary glioblastoma. J. Neurooncol.. *129*, 495-503.
18. Zhang, H., Cong, Q.X., Zhang, S.G., Zhai, X.W., Li, H.F., and Li, S.Q. (2018). High Expression Levels of Fascin-1 Protein in Human Gliomas and its Clinical Relevance. Open Med (Wars). *13*, 544-550.
19. Gunal, A., Onguru, O., Safali, M., and Beyzadeoglu, M. (2008). Fascin expression [corrected] in glial tumors and its prognostic significance in glioblastomas. Neuropathology. *28*, 382-386.
20. Gao, Z., Zhang, Q., Kong, F., Chen, G., Li, M., Guo, H., Liang, J., Bao, Y., and Ling, F. (2012). Fascin expression in skull base chordoma: correlation with tumor recurrence and dura erosion. Med. Oncol.. *29*, 2438-2444.
21. Yoder, B.J., Tso, E., Skacel, M., Pettay, J., Tarr, S., Budd, T., Tubbs, R.R., Adams, J.C., and Hicks, D.G. (2005). The expression of fascin, an actin-bundling motility protein, correlates with hormone receptor-negative breast cancer and a more aggressive clinical course. Clin. Cancer Res.. *11*, 186-192.
22. Wang, C.Q., Li, Y., Huang, B.F., Zhao, Y.M., Yuan, H., Guo, D., Su, C.M., Hu, G.N., Wang, Q., Long, T., et al. (2017). EGFR conjunct FSCN1 as a Novel Therapeutic Strategy in Triple-Negative Breast Cancer. Sci Rep. *7*, 15654.
23. Min, K.W., Kim, D.H., Do, S.I., Chae, S.W., Kim, K., Sohn, J.H., Pyo, J.S., Lee, H.J., Kim, D.H., Oh, S., et al. (2016). Negative association between GATA3 and fascin could predict relapse-free and overall survival in patients with breast cancer. Virchows Arch.. *468*, 409-416.
24. Youssef, N.S., and Hakim, S.A. (2014). Association of Fascin and matrix metalloproteinase-9 expression with poor prognostic parameters in breast carcinoma of Egyptian women. Diagn Pathol. *9*, 136.
25. Omran, O.M., and Al Sheeha, M. (2015). Cytoskeletal Focal Adhesion Proteins Fascin-1 and Paxillin Are Predictors of Malignant Progression and Poor Prognosis in Human Breast Cancer. J. Environ. Pathol. Toxicol. Oncol.. *34*, 201-212.
26. Min, K.W., Chae, S.W., Kim, D.H., DO, S.I., Kim, K., Lee, H.J., Sohn, J.H., Pyo, J.S., Kim, D.H., Oh, S., et al. (2015). Fascin expression predicts an aggressive clinical course in patients with advanced breast cancer. Oncol Lett. *10*, 121-130.
27. Lee, H.J., An, H.J., Kim, T.H., Kim, G., Kang, H., Heo, J.H., Kwon, A.Y., and Kim, S. (2017). Fascin expression is inversely correlated with breast cancer metastasis suppressor 1 and predicts a worse survival outcome in node-negative breast cancer patients. J Cancer. *8*, 3122-3129.
28. Rodríguez-Pinilla, S.M., Sarrió, D., Honrado, E., Hardisson, D., Calero, F., Benitez, J., and Palacios, J. (2006). Prognostic significance of basal-like phenotype and fascin expression in node-negative invasive breast carcinomas. Clin. Cancer Res.. *12*, 1533-1539.
29. Esnakula, A.K., Ricks-Santi, L., Kwagyan, J., Kanaan, Y.M., DeWitty, R.L., Wilson, L.L., Gold, B., Frederick, W.A., and Naab, T.J. (2014). Strong association of fascin expression with triple negative breast cancer and basal-like phenotype in African-American women. J. Clin. Pathol.. *67*, 153-160.
30. Wang, C.Q., Tang, C.H., Chang, H.T., Li, X.N., Zhao, Y.M., Su, C.M., Hu, G.N., Zhang, T., Sun, X.X., Zeng, Y., et al. (2016). Fascin-1 as a novel diagnostic marker of triple-negative breast cancer. Cancer Med. *5*, 1983-1988.
31. Kabukcuoglu, S., Ozalp, S.S., Oner, U., Açikalin, M.F., Yalcin, O.T., and Colak, E. (2005). Fascin, an actin-

bundling protein expression in cervical neoplasms. Eur. J. Gynaecol. Oncol.. *26*, 636-641.

32. Stewart, C.J., Crook, M., and Loi, S. (2012). Fascin expression in endocervical neoplasia: correlation with tumour morphology and growth pattern. J. Clin. Pathol.. *65*, 213-217.
33. Koay, M.H., Crook, M., and Stewart, C.J. (2014). Fascin expression in cervical normal squamous epithelium, cervical intraepithelial neoplasia, and superficially invasive (stage IA1) squamous carcinoma of the cervix. Pathology. *46*, 433-438.
34. Hashimoto, Y., Skacel, M., Lavery, I.C., Mukherjee, A.L., Casey, G., and Adams, J.C. (2006). Prognostic significance of fascin expression in advanced colorectal cancer: an immunohistochemical study of colorectal adenomas and adenocarcinomas. BMC Cancer. *6*, 241.
35. Tsai, W.C., Chao, Y.C., Sheu, L.F., Chang, J.L., Nieh, S., and Jin, J.S. (2007). Overexpression of fascin-1 in advanced colorectal adenocarcinoma: tissue microarray analysis of immunostaining scores with clinicopathological parameters. Dis. Markers. *23*, 153-160.
36. Qualtrough, D., Singh, K., Banu, N., Paraskeva, C., and Pignatelli, M. (2009). The actin-bundling protein fascin is overexpressed in colorectal adenomas and promotes motility in adenoma cells in vitro. Br. J. Cancer. *101*, 1124-1129.
37. Chan, C., Jankova, L., Fung, C.L., Clarke, C., Robertson, G., Chapuis, P.H., Bokey, L., Lin, B.P., Dent, O.F., and Clarke, S. (2010). Fascin expression predicts survival after potentially curative resection of node-positive colon cancer. Am. J. Surg. Pathol.. *34*, 656-666.
38. Oh, S.Y., Kim, Y.B., Suh, K.W., Paek, O.J., and Moon, H.Y. (2012). Prognostic impact of fascin-1 expression is more significant in advanced colorectal cancer. J. Surg. Res.. *172*, 102-108.
39. Puppa, G., Maisonneuve, P., Sonzogni, A., Masullo, M., Chiappa, A., Valerio, M., Zampino, M.G., Franceschetti, I., Capelli, P., Chilosi, M., et al. (2007). Independent prognostic value of fascin immunoreactivity in stage III-IV colonic adenocarcinoma. Br. J. Cancer. *96*, 1118-1126.
40. Jung, E.J., Lee, J.H., Min, B.W., Kim, Y.S., and Choi, J.S. (2011). Clinicopathologic significance of fascin, extracellular matrix metalloproteinase inducer, and ezrin expressions in colorectal adenocarcinoma. Indian J Pathol Microbiol. *54*, 32-36.
41. Piskor, B.M., Pryczynicz, A., Lubowicka, E., Miniewska, K., Zinczuk, J., Zareba, K., and Guzinska-Ustymowicz, K. (2018). Immunohistochemical expression of Fascin-1 in colorectal cancer in relation to clinical and pathological parameters. Folia Histochem. Cytobiol.. *1*, 106-112.
42. Ozerhan, I.H., Ersoz, N., Onguru, O., Ozturk, M., Kurt, B., and Cetiner, S. (2010). Fascin expression in colorectal carcinomas. Clinics (Sao Paulo). *65*, 157-164.
43. Binnetoglu, A., Sari, M., Baglam, T., Erbarut Seven, I., Yumusakhuylu, A.C., Topuz, M.F., and Batman, C. (2015). Fascin expression in cholesteatoma: correlation with destruction of the ossicular chain and extent of disease. Clin Otolaryngol. *40*, 335-340.
44. Onder, S., Taskin, O.C., Sen, F., Topuz, S., Kucucuk, S., Sozen, H., Ilhan, R., Tuzlali, S., and Yavuz, E. (2017). High expression of SALL4 and fascin, and loss of E-cadherin expression in undifferentiated/dedifferentiated carcinomas of the endometrium: An immunohistochemical and clinicopathologic study. Medicine (Baltimore). *96*, e6248.
45. Gun, B.D., Bahadir, B., Bektas, S., Barut, F., Yurdakan, G., Kandemir, N.O., and Ozdamar, S.O. (2012). Clinicopathological significance of fascin and CD44v6 expression in endometrioid carcinoma. Diagn Pathol. *7*, 80.
46. Kabukcuoglu, S., Oner, U., Ozalp, S.S., Dundar, E., Yalcin, O.T., and Colak, E. (2006). Prognostic significance of fascin expression in endometrioid carcinoma. Eur. J. Gynaecol. Oncol.. *27*, 481-486.
47. Stewart, C.J., and Crook, M.L. (2015). Fascin expression in undifferentiated and dedifferentiated endometrial carcinoma. Hum. Pathol.. *46*, 1514-1520.
48. Akanuma, N., Hoshino, I., Akutsu, Y., Murakami, K., Isozaki, Y., Maruyama, T., Yusup, G., Qin, W., Toyozumi,

T., Takahashi, M., et al. (2014). MicroRNA-133a regulates the mRNAs of two invadopodia-related proteins, FSCN1 and MMP14, in esophageal cancer. Br. J. Cancer. *110*, 189-198.

49. Takikita, M., Hu, N., Shou, J.Z., Giffen, C., Wang, Q.H., Wang, C., Hewitt, S.M., and Taylor, P.R. (2011). Fascin and CK4 as biomarkers for esophageal squamous cell carcinoma. Anticancer Res.. *31*, 945-952.
50. Zhao, Q., Shen, J.H., Shen, Z.Y., Wu, Z.Y., Xu, X.E., Xie, J.J., Wu, J.Y., Huang, Q., Lu, X.F., Li, E.M., et al. (2010). Phosphorylation of fascin decreases the risk of poor survival in patients with esophageal squamous cell carcinoma. J. Histochem. Cytochem.. *58*, 979-988.
51. Zhang, H., Xu, L., Xiao, D., Xie, J., Zeng, H., Cai, W., Niu, Y., Yang, Z., Shen, Z., and Li, E. (2006). Fascin is a potential biomarker for early-stage oesophageal squamous cell carcinoma. J. Clin. Pathol.. *59*, 958-964.
52. Hashimoto, Y., Ito, T., Inoue, H., Okumura, T., Tanaka, E., Tsunoda, S., Higashiyama, M., Watanabe, G., Imamura, M., and Shimada, Y. (2005). Prognostic significance of fascin overexpression in human esophageal squamous cell carcinoma. Clin. Cancer Res.. *11*, 2597-2605.
53. Yamamoto, H., Kohashi, K., Fujita, A., and Oda, Y. (2013). Fascin-1 overexpression and miR-133b downregulation in the progression of gastrointestinal stromal tumor. Mod. Pathol.. *26*, 563-571.
54. Ozcan, A., Karslioğlu, Y., Günal, A., Cermık, A.H., Kurt, B., and Ongürü, O. (2011). Fascin expression and its potential significance in gastrointestinal stromal tumors. Turk J Gastroenterol. *22*, 363-368.
55. Wu, D., Chen, L., Liao, W., Ding, Y., Zhang, Q., Li, Z., and Liu, L. (2010). Fascin1 expression predicts poor prognosis in patients with nasopharyngeal carcinoma and correlates with tumor invasion. Ann. Oncol.. *21*, 589-596.
56. Papaspyrou, K., Brochhausen, C., Schmidtmann, I., Fruth, K., Gouveris, H., Kirckpatrick, J., Mann, W., and Brieger, J. (2014). Fascin upregulation in primary head and neck squamous cell carcinoma is associated with lymphatic metastasis. Oncol Lett. *7*, 2041-2046.
57. Routray, S., Kheur, S., Chougule, H.M., Mohanty, N., and Dash, R. (2017). Establishing Fascin over-expression as a strategic regulator of neoplastic aggression and lymph node metastasis in oral squamous cell carcinoma tumor microenvironment. Ann Diagn Pathol. *30*, 36-41.
58. Gao, W., Zhang, C., Feng, Y., Chen, G., Wen, S., Huangfu, H., and Wang, B. (2012). Fascin-1, ezrin and paxillin contribute to the malignant progression and are predictors of clinical prognosis in laryngeal squamous cell carcinoma. PLoS ONE. *7*, e50710.
59. Durmaz, A., Kurt, B., Ongoru, O., Karahatay, S., Gerek, M., and Yalcin, S. (2010). Significance of fascin expression in laryngeal squamous cell carcinoma. J Laryngol Otol. *124*, 194-198.
60. Zou, J., Yang, H., Chen, F., Zhao, H., Lin, P., Zhang, J., Ye, H., Wang, L., and Liu, S. (2010). Prognostic significance of fascin-1 and E-cadherin expression in laryngeal squamous cell carcinoma. Eur. J. Cancer Prev.. *19*, 11-17.
61. Chen, S.F., Yang, S.F., Li, J.W., Nieh, P.C., Lin, S.Y., Fu, E., Bai, C.Y., Jin, J.S., Lin, C.Y., and Nieh, S. (2007). Expression of fascin in oral and oropharyngeal squamous cell carcinomas has prognostic significance - a tissue microarray study of 129 cases. Histopathology. *51*, 173-183.
62. Lee, T.K., Poon, R.T., Man, K., Guan, X.Y., Ma, S., Liu, X.B., Myers, J.N., and Yuen, A.P. (2007). Fascin over-expression is associated with aggressiveness of oral squamous cell carcinoma. Cancer Lett.. *254*, 308-315.
63. Alam, H., Bhate, A.V., Gangadaran, P., Sawant, S.S., Salot, S., Sehgal, L., Dange, P.P., Chaukar, D.A., D'cruz, A.K., Kannanl, S., et al. (2012). Fascin overexpression promotes neoplastic progression in oral squamous cell carcinoma. BMC Cancer. *12*, 32.
64. Chen, Y., Tian, T., Li, Z.Y., Wang, C.Y., Deng, R., Deng, W.Y., Yang, A.K., Chen, Y.F., and Li, H. (2019). FSCN1 is an effective marker of poor prognosis and a potential therapeutic target in human tongue squamous cell carcinoma. Cell Death Dis. *10*, 356.
65. Gu, M.J., Kim, J.Y., and Park, J.B. (2014). Fascin expression predicts lymph node metastasis and worse survival in small intestinal carcinoma. Pathology. *46*, 21-24.

66. Zhang, M., Zhao, Z., Duan, X., Chen, P., Peng, Z., and Qiu, H. (2018). FSCN1 predicts survival and is regulated by a PI3K-dependent mechanism in renal cell carcinoma. J. Cell. Physiol.. *233*, 4748-4758.
67. Huang, W., Cen, S., Kang, X.L., Wang, W.F., Wang, Y., and Chen, X. (2016). TGF-β1-induced Fascin1 promotes cell invasion and metastasis of human 786-0 renal carcinoma cells. Acta Histochem.. *118*, 144-151.
68. Tsai, W.C., Sheu, L.F., Nieh, S., Yu, C.P., Sun, G.H., Lin, Y.F., Chen, A., and Jin, J.S. (2007). Association of EMMPRIN and fascin expression in renal cell carcinoma: correlation with clinicopathological parameters. World J Urol. *25*, 73-80.
69. Zigeuner, R., Droschl, N., Tauber, V., Rehak, P., and Langner, C. (2006). Biologic significance of fascin expression in clear cell renal cell carcinoma: systematic analysis of primary and metastatic tumor tissues using a tissue microarray technique. Urology. *68*, 518-522.
70. Jin, J.S., Yu, C.P., Sun, G.H., Lin, Y.F., Chiang, H., Chao, T.K., Tsai, W.C., and Sheu, L.F. (2006). Increasing expression of fascin in renal cell carcinoma associated with clinicopathological parameters of aggressiveness. Histol. Histopathol.. *21*, 1287-1293.
71. Iguchi, T., Aishima, S., Umeda, K., Sanefuji, K., Fujita, N., Sugimachi, K., Gion, T., Taketomi, A., Maehara, Y., and Tsuneyoshi, M. (2009). Fascin expression in progression and prognosis of hepatocellular carcinoma. J Surg Oncol. *100*, 575-579.
72. Wang, G., Zhu, S., Gu, Y., Chen, Q., Liu, X., and Fu, H. (2015). MicroRNA-145 and MicroRNA-133a Inhibited Proliferation, Migration, and Invasion, While Promoted Apoptosis in Hepatocellular Carcinoma Cells Via Targeting FSCN1. Dig. Dis. Sci.. *60*, 3044-3052.
73. Huang, X., Ji, J., Xue, H., Zhang, F., Han, X., Cai, Y., Zhang, J., and Ji, G. (2012). Fascin and cortactin expression is correlated with a poor prognosis in hepatocellular carcinoma. Eur J Gastroenterol Hepatol. *24*, 633-639.
74. Hayashi, Y., Osanai, M., and Lee, G.H. (2011). Fascin-1 expression correlates with repression of E-cadherin expression in hepatocellular carcinoma cells and augments their invasiveness in combination with matrix metalloproteinases. Cancer Sci.. *102*, 1228-1235.
75. Zhao, W., Gao, J., Wu, J., Liu, Q.H., Wang, Z.G., Li, H.L., and Xing, L.H. (2015). Expression of Fascin-1 on human lung cancer and paracarcinoma tissue and its relation to clinicopathological characteristics in patients with lung cancer. Onco Targets Ther. *8*, 2571-2576.
76. Zhang, Y., Liang, B., and Dong, H. (2018). Expression of fascin_1 protein in cancer tissues of patients with nonsmall cell lung cancer and its relevance to patients' clinicopathologic features and prognosis. J Cancer Res Ther. *14*, 856-859.
77. Yang, L., Teng, Y., Han, T.P., Li, F.G., Yue, W.T., and Wang, Z.T. (2017). Clinical significance of fascin-1 and laminin-5 in non-small cell lung cancer. Genet. Mol. Res.. *16*.
78. Zhang, J., Wang, X., Zhang, Y., Wu, J., and Zhou, N. (2016). Leucine-rich repeats and immunoglobulin-like domains protein 1 and fascin actin-bundling protein 1 expression in nonsmall cell lung cancer. J Cancer Res Ther. *12*, C248-248C251.
79. Liang, Z., Wang, Y., Shen, Z., Teng, X., Li, X., Li, C., Wu, W., Zhou, Z., and Wang, Z. (2016). Fascin 1 promoted the growth and migration of non-small cell lung cancer cells by activating YAP/TEAD signaling. Tumour Biol.. *37*, 10909-10915.
80. Luo, A., Yin, Y., Li, X., Xu, H., Mei, Q., and Feng, D. (2015). The clinical significance of FSCN1 in non-small cell lung cancer. Biomed. Pharmacother.. *73*, 75-79.
81. Ling, X.L., Zhang, T., Hou, X.M., and Zhao, D. (2015). Clinicopathological significance of fascin-1 expression in patients with non-small cell lung cancer. Onco Targets Ther. *8*, 1589-1595.
82. Choi, P.J., Yang, D.K., Son, C.H., Lee, K.E., Lee, J.I., and Roh, M.S. (2006). Fascin immunoreactivity for preoperatively predicting lymph node metastases in peripheral adenocarcinoma of the lung 3 cm or less in diameter. Eur J Cardiothorac Surg. *30*, 538-542.

83. Pelosi, G., Pasini, F., Fraggetta, F., Pastorino, U., Iannucci, A., Maisonneuve, P., Arrigoni, G., De Manzoni, G., Bresaola, E., and Viale, G. (2003). Independent value of fascin immunoreactivity for predicting lymph node metastases in typical and atypical pulmonary carcinoids. Lung Cancer. *42*, 203-213.
84. Pelosi, G., Pastorino, U., Pasini, F., Maissoneuve, P., Fraggetta, F., Iannucci, A., Sonzogni, A., De Manzoni, G., Terzi, A., Durante, E., et al. (2003). Independent prognostic value of fascin immunoreactivity in stage I nonsmall cell lung cancer. Br. J. Cancer. *88*, 537-547.
85. Alici, O., Kefeli, M., Yildiz, L., Baris, S., Karagoz, F., and Kandemir, B. (2014). Fascin and EMMPRIN expression in primary mucinous tumors of ovary: a tissue microarray study. Pathol. Res. Pract.. *210*, 934-938.
86. Wen, Y.H., Yee, H., Goswami, S., and Shukla, P.S. (2009). Fascin expression in serous tumors of ovary correlates with aggressiveness of malignancy. Int. J. Gynecol. Pathol.. *28*, 187-192.
87. Lin, C.K., Su, H.Y., Tsai, W.C., Sheu, L.F., and Jin, J.S. (2008). Association of cortactin, fascin-1 and epidermal growth factor receptor (EGFR) expression in ovarian carcinomas: correlation with clinicopathological parameters. Dis. Markers. *25*, 17-26.
88. Daponte, A., Kostopoulou, E., Papandreou, C.N., Daliani, D.D., Minas, M., Koukoulis, G., and Messinis, I.E. (2008). Prognostic significance of fascin expression in advanced poorly differentiated serous ovarian cancer. Anticancer Res.. *28*, 1905-1910.
89. El-Balat, A., Arsenic, R., Sänger, N., Karn, T., Becker, S., Holtrich, U., and Engels, K. (2016). Fascin-1 expression as stratification marker in borderline epithelial tumours of the ovary. J. Clin. Pathol.. *69*, 142-148.
90. Hanker, L.C., Karn, T., Holtrich, U., Graeser, M., Becker, S., Reinhard, J., Ruckhäberle, E., Gevensleben, H., and Rody, A. (2013). Prognostic impact of fascin-1 (FSCN1) in epithelial ovarian cancer. Anticancer Res.. *33*, 371-377.
91. Lin, C.K., Chao, T.K., Yu, C.P., Yu, M.H., and Jin, J.S. (2009). The expression of six biomarkers in the four most common ovarian cancers: correlation with clinicopathological parameters. APMIS. *117*, 162-175.
92. Park, S.H., Song, J.Y., Kim, Y.K., Heo, J.H., Kang, H., Kim, G., An, H.J., and Kim, T.H. (2014). Fascin1 expression in high-grade serous ovarian carcinoma is a prognostic marker and knockdown of fascin1 suppresses the proliferation of ovarian cancer cells. Int. J. Oncol.. *44*, 637-646.
93. Yamaguchi, H., Inoue, T., Eguchi, T., Miyasaka, Y., Ohuchida, K., Mizumoto, K., Yamada, T., Yamaguchi, K., Tanaka, M., and Tsuneyoshi, M. (2007). Fascin overexpression in intraductal papillary mucinous neoplasms (adenomas, borderline neoplasms, and carcinomas) of the pancreas, correlated with increased histological grade. Mod. Pathol.. *20*, 552-561.
94. Tsai, W.C., Chao, Y.C., Sheu, L.F., Lin, Y.F., Nieh, S., Chen, A., Yu, C.P., and Jin, J.S. (2007). EMMPRIN and fascin overexpression associated with clinicopathologic parameters of pancreatobiliary adenocarcinoma in Chinese people. APMIS. *115*, 929-938.
95. Liu, C., Gao, H., Cao, L., Gui, S., Liu, Q., Li, C., Li, D., Gong, L., and Zhang, Y. (2016). The role of FSCN1 in migration and invasion of pituitary adenomas. Mol. Cell. Endocrinol.. *419*, 217-224.
96. Darnel, A.D., Behmoaram, E., Vollmer, R.T., Corcos, J., Bijian, K., Sircar, K., Su, J., Jiao, J., Alaoui-Jamali, M.A., and Bismar, T.A. (2009). Fascin regulates prostate cancer cell invasion and is associated with metastasis and biochemical failure in prostate cancer. Clin. Cancer Res.. *15*, 1376-1383.
97. Ma, Y., Faller, W.J., Sansom, O.J., Brown, E.R., Doig, T.N., Melton, D.W., and Machesky, L.M. (2015). Fascin expression is increased in metastatic lesions but does not correlate with progression nor outcome in melanoma. Melanoma Res.. *25*, 169-172.
98. Goncharuk, V.N., Ross, J.S., and Carlson, J.A. (2002). Actin-binding protein fascin expression in skin neoplasia. J. Cutan. Pathol.. *29*, 430-438.
99. Valkov, A., Sorbye, S.W., Kilvaer, T.K., Donnem, T., Smeland, E., Bremnes, R.M., and Busund, L.T. (2011). The prognostic impact of TGF-β1, fascin, NF-κB and PKC-ζ expression in soft tissue sarcomas. PLoS ONE. *6*, e17507.

100. Tsai, W.C., Jin, J.S., Chang, W.K., Chan, D.C., Yeh, M.K., Cherng, S.C., Lin, L.F., Sheu, L.F., and Chao, Y.C. (2007). Association of cortactin and fascin-1 expression in gastric adenocarcinoma: correlation with clinicopathological parameters. J. Histochem. Cytochem.. *55*, 955-962.
101. Tu, L., Xu, J., Wang, M., Zhao, W.Y., Zhang, Z.Z., Zhu, C.C., Tang, D.F., Zhang, Y.Q., Wang, D.H., Zuo, J., et al. (2016). Correlations of fascin-1 and cadherin-17 protein expression with clinicopathologic features and prognosis of patients with gastric cancer. Tumour Biol.. *37*, 8775-8782.
102. Kim, S.J., Kim, D.C., Kim, M.C., Jung, G.J., Kim, K.H., Jang, J.S., Kwon, H.C., Kim, Y.M., and Jeong, J.S. (2012). Fascin expression is related to poor survival in gastric cancer. Pathol. Int.. *62*, 777-784.
103. Hashimoto, Y., Shimada, Y., Kawamura, J., Yamasaki, S., and Imamura, M. (2004). The prognostic relevance of fascin expression in human gastric carcinoma. Oncology. *67*, 262-270.
104. Chen, G., Zhang, F.R., Ren, J., Tao, L.H., Shen, Z.Y., Lv, Z., Yu, S.J., Dong, B.F., Xu, L.Y., and Li, E.M. (2008). Expression of fascin in thyroid neoplasms: a novel diagnostic marker. J. Cancer Res. Clin. Oncol.. *134*, 947-951.
105. Kefeli, M., Yildiz, L., Kaya, F.C., Aydin, O., and Kandemir, B. (2009). Fascin expression in uterine smooth muscle tumors. Int. J. Gynecol. Pathol.. *28*, 328-333.
106. Richmond, A.M., Blake, E.A., Torkko, K., Smith, E.E., Spillman, M.A., and Post, M.D. (2017). Fascin Is Associated With Aggressive Behavior and Poor Outcome in Uterine Carcinosarcoma. Int. J. Gynecol. Cancer. *27*, 1895-1903.
