# Supplementary material for: Fascin actin-bundling protein 1 in human cancer: promising biomarker or therapeutic target?
Source: Mol Ther Oncolytics. 2021 Jan 20;20:240–64. doi: 10.1016/j.omto.2020.12.014 (PMC7873579; doi:10.1016/j.omto.2020.12.014)
Supplement: Table S2. Clinicopathological and prognostic significance of tissue and serum FSCN1 levels in different human cancers [file mmc2.pdf]

Table S2: Clinicopathological and prognostic significance of tissue and serum FSCN1 levels in different human cancers

Cancer types	Sample size/type	Association with clinical pathological parameters	Outcome of patients	Statistical method	Independent prognostic indicator?	Refs
Adrenocortical Carcinoma	37 patients/tissue	Nd	Poor DFS (P=0.043) and OS (P=0.024)	Kaplan-Meier analysis	Yes	1
Adrenocortical Carcinoma	51 patients/tissue	ENSAT tumor stage (p = 0.04)	Worse OS (P=0.001) and DFS (P=0.009)	Cox regression analysis	Yes	2
Bladder urothelial carcinoma	111 patients/tissue	Tumor size (P=0.011) and pT stage (P=0.001)	Lower RFS (P=0.000)	Kaplan-Meier and Cox regression analysis	Yes	3
Bladder urothelial carcinoma	122 patients/tissue	Older patients (P=0.005) and local disease recurrence (P=0.002)	Poor OS (P=0.027)	Kaplan-Meier analysis	Nd	4
Bladder cancer	125 patients/tissue	Tumor pathological (pT) stage (P<0.001)	Nd	Nd	Nd	5
Bladder cancer	88 patients/tissue	Histological grade (P=0.024) and pT stage (P<0.001)	Nd	Nd	Nd	6
Breast cancer	127 patients/tissue	Triple negative (P<0.0001), high tumor grade (P = 0.002) and high expression of Ki-67 (P = 0.004)	Worse 5-year PFS (P=0.032)	Kaplan-Meier and multivariate Cox regression analysis	Nd	7
Breast cancer	61 patients/tissue	High grade histology, lymph node involvement，larger tumor size (p = 0.04)	Nd	Nd	Nd	8
Breast cancer (triple negative breast cancer)	202 patients/tissue	TN subtype, ER negativity, PR negativity, EN grade 3 (all P<0.0001)	Decreased OS (P=0.003)	Kaplan-Meier and Cox regression analysis	Yes	9
Breast cancer	183 patients/tissue	Negative nodal metastasis, higher histological grade, higher nuclear grade, ER/PR/HER2 negativity, and triple-negative subtype (all P<0.05).	Shorter DFS (P=0.005) and OS (P=0.020)	Kaplan-Meier and Cox regression analysis	Yes	10
Breast cancer	139 chemo-treated patients/tissue	Nd	Poor DFS (P=0.0098) and OS (P=0.0026)	Kaplan-Meier analysis	Nd	11
Breast cancer (invasive ductal carcinoma)	239 patients/tissue	High histological grade, presence of tumor necrosis, negativity of ER and PR, and high p53 expression (all P<0.05)	Shorter OS (P=0.009)	Kaplan-Meier and multivariate Cox regression analysis	Nd	12

Breast cancer (invasive ductal carcinoma)	194 patients/tissue	High histological grade, tumor necrosis, resistance to adjuvant therapy, high expression of p53 and Ki-67 and ER/PR negativity (all P<0.05)	Poor DFS and OS (all P<0.05)	Kaplan-Meier and Cox regression analysis	Nd	13
Breast cancer (invasive ductal carcinoma)	467 patients/tissue	Tumor size (P=0.024), grade (P<0.0001), stage (P=0.045), ER/PR negativity (P<0.0001), and axillary lymph node metastasis (P=0.024)	Worse RFS (P=0.0073) and OS (P=0.0089)	Kaplan-Meier analysis	Nd	14
Breast cancer	100 patients/tissue	Tumor grade, clinical stage, lymph-node metastasis grade, and HER2 expression (all P<0.05)	Nd	Nd	Nd	15
Breast cancer	67 patients/tissue	Lymph node metastases (P=0.001), advanced tumor stage (P=0.004), ER negativity (P=0.002), and PR negativity (P=0.001)	Nd	Nd	Nd	16
Breast cancer	210 patients/tissue	ER negativity, PR negativity, Bloom-Richardson grade 3 (each P<0.001) and advanced stage (P=0.04)	Decreased mean DFS and mean OS (P=0.002)	Kaplan-Meier analysis	Yes	17
Breast cancer	71 patients/tissue	Tumor size (P=0.035), basal-like phenotype (P<0.001), ER negative (P<0.001), PR negative (P=0.02), local and systematic metastasis (P=0.017)	Shorter DFS (P=0.0007)	Kaplan-Meier analysis	Nd	18
Cholangiocarcinoma	142 patients/tissue	Tumor dedifferentiation, venous invasion and lymph node or distant metastasis (all, P<0.05)	Shorter median survival (P<0.001)	Kaplan-Meier and Cox regression analysis	Yes	19
Intrahepatic cholangiocarcinoma	84 patients/tissue	Poorly differentiated (P=0.0019)	Poor OS (P=0.0085)	Kaplan-Meier and multivariate Cox regression analysis	Yes	20
Cholesteatoma	28 patients/tissue	Destruction of ossicular chain and extent of the disease (all, P<0.05)	Nd	Nd	Nd	21
Colonic adenocarcinoma	228 patients/tissue	Sex, tumor grade and stage, mucinous differentiation, number of metastatic lymph nodes, extranodal tumor extension, and the occurrence of distant metastases (each P<0.05)	Shorter DFS and OS (each P<0.05)	Kaplan-Meier and Cox regression analysis	Yes	22

Colorectal adenocarcinomas	142 patients/tissue	Tumor stage (P=0.01), increasing age (P=0.035) and lymph node metastasis (P=0.03)	Poor prognosis for stage III/IV adenocarcinoma patients (P=0.023)	Kaplan-Meier and Cox regression analysis	Yes	23
Colorectal cancer	126 patients/tissue	Distant metastases (P=0.028)	Lower DFS (P=0.028) and OS (P=0.041)	Kaplan-Meier and multivariate Cox regression analysis	Yes	24
Colorectal cancer	51 patients/tissue	Histological type (P=0.012), infiltration of cancer cells to blood and lymphatic vessels (P=0.038)	Nd	Nd	Nd	25
Colorectal cancer	GSE21510/TCGA	Nd	Reduced OS (P=0.0044) and DFS (P=0.055)	Kaplan-Meier analysis	Nd	26
Colorectal cancer	466 patients/tissue	Tumor site and size, direct spread, histologic type and grade, and Z4 nodes involved (all p<0.001)	Diminished survival (P=0.007)	Kaplan-Meier and Cox regression analysis	Nd	27
Colorectal cancer	167 patients/tissue	Lymph node metastasis (P=0.002) and advanced stage presentation (P=0.007)	Shorter two-year survival (P=0.003)	Kaplan-Meier analysis	Nd	28
Colorectal cancer	94 patients/tissue	Tumor size, TNM stage and distant metastasis (P < 0.05)	Reduced OS (P<0.001)	Kaplan–Meier analysis	Nd	29
Colorectal cancer	210 patients/tissue	Advanced tumor depth (P=0.035)	poor OS (P=0.016)	Kaplan-Meier and Cox regression analysis	Yes	30
Endometrioid carcinomas	47 patients/tissue	Tumor grade (P=0.003) and neural invasion (P=0.036)	Nd	Nd	Nd	31
Esophageal squamous cell carcinoma	149 patients and 98 control /serum	Early detection of ESCC (P< 0.05), age (P < 0.05)	Nd	Nd	Nd	32
Esophageal squamous cell carcinoma	80 patients/tissue	Pathological grade (P=0.004), TNM stage (T stage, P=0.002; N stage, P=0.000) and clinical stage (P=0.004)	Reduced OS (P<0.001)	Kaplan-Meier and Cox regression analysis	Yes	33
Esophageal squamous cell carcinoma	231 patients/tissue	Tumor grade (P<0.01)	Not correlate with patient survival	Kaplan-Meier analysis	No	34
Esophageal squamous cell carcinoma	254 patients/tissue	Tumor size (P=0.001)	Worse OS (P=0.006)	Kaplan-Meier and multivariate Cox regression analysis	Yes	35

Esophageal squamous cell carcinoma	200 patients/tissue	Extent of the tumor (P=0.002) and lymph node metastasis (P=0.003)	Lower survival rate (p<0.05)	Kaplan-Meier and multivariate Cox regression analysis	Yes	36
Extrahepatic bile duct carcinomas	114 patients/tissue	Histological grade (P<0.0001), primary tumor (T) (P=0.002), TNM stage (P=0.036), lymphatic invasion (P=0.048), venous invasion (P=0.024), and adjacent organ invasion (P<0.0001).	Poor OS (P=0.0001)	Kaplan-Meier and Cox regression analysis	Yes	37
Gallbladder cancer	43 patients/tissue	Poorer differentiation, deeper invasion depth, lymph node metastasis, a higher American Joint Committee on Cancer stage, and recurrence (each P<0.05)	Shorter survival periods (P=0.001)	Kaplan-Meier and multivariate Cox regression analysis	No	38
Gastric adenocarcinomas	100 patients/tissue	Histological grading (P<0.05)	Worse survival (P>0.05)	Kaplan-Meier analysis	Nd	39
Gastric adenocarcinomas	285 patients/tissue	Old age, advanced T and N category, large tumor size, high histological grade, lymphatic and vascular invasion (all P<0.05)	Poor DFS and OS (all P<0.05)	Kaplan-Meier and Cox regression analysis	Nd	40
Gastric cancer	1105 patients/ meta-analysis	Lymph node metastasis, TNM staging (all P<0.05)	Poor OS and PFS (all P<0.05)	Kaplan-Meier analysis	Nd	41
Gastric cancer	204 patients/tissue	Tumor size (P=0.001) and lauren classification (P=0.001)	Independent risk factor (P<0.05)	Kaplan-Meier and multivariate Cox regression analysis	Yes	42
Gastric cancer	471 patients/tissue	High clinical stage (P<0.001), high T stage (P<0.001), nodal metastasis (P<0.001), lymphovascular invasion (P=0.001) and the intestinal type of Lauren classification (P=0.015)	Worse survival rates (P<0.001)	Kaplan-Meier and multivariate Cox regression analysis	Yes	43
Gastric cancer	214 patients/tissue	Age (P=0.005), serosal invasion (P=0.013), positive lymph node metastasis (P=0.006), histopathological grading (P=0.019), TNM stage (P=0.003) and recurrence (P=0.006)	Lower survival rate (P=0.029)	Kaplan-Meier and multivariate Cox regression analysis	No	44

Gastrointestinal stromal tumor	147 patients/tissue	Tumor size, mitotic counts, risk grade, blood vessel invasion and mucosal ulceration (all P<0.0001)	Shorter DFS (P<0.0001)	Kaplan-Meier and Cox regression analysis	No	45
Glioblastoma	37 patients/tissue	No statistically significant differences	Shorter PFS and OS (all P<0.05)	Kaplan-Meier and Cox regression analysis	Yes	46
Glioma	120 patients/tissue	WHO tumor grading (P=0.001)	Poorer PFS (P =0.021) and OS (P=0.012)	Kaplan-Meier and Cox regression analysis	Yes	47
Glial tumors (various cancer samples)	76 patients/tissue	Histologic grade (P=0.002)	Poor OS (P=0.040)	Kaplan-Meier and multivariate Cox regression analysis	Yes	48
Head and neck cancer	40 patients/serum	Pathological lymph node metastasis (p=0.022)	Nd	Nd	Nd	49
Head and neck squamous cell carcinoma	25 patients/tissue	Lymph node metastases (P=0.03)	Not significantly correlated with OS, RFS and event-free survival (P>0.05)	Kaplan-Meier analysis	No	50
Hepatocellular carcinoma	77 patients/tissue	Histological differentiation (P=0.001) and metastasis (P=0.008)	Shorter median survival (P=0.047)	Kaplan-Meier analysis	Nd	51
Hepatocellular carcinoma	137 patients/tissue	Tumor size (P=0.0239), histological differentiation (P=0.0018), portal venous invasion (P=0.0029), bile duct invasion (P=0.0333), intrahepatic metastasis (P=0.0403)	Poorer DFS (P =0.0221) and OS (P=0.0076)	Kaplan-Meier and multivariate Cox regression analysis	Yes	52
Laryngeal squamous cell carcinoma	216 patients/tissue	Poor tumor differentiation, cervical lymph node metastasis (N+), and advanced clinical stage (III+IV), advanced tumor stage (T3+T4) (all P<0.05)	Poor OS (P=0.002)	Kaplan-Meier and multivariate Cox regression analysis	Yes	53
Laryngeal squamous cell carcinoma	30 patients/tissue	Tumor stage (P=0.022), node stage (P=0.024) and clinical stage (P=0.014)	Nd	Nd	Nd	54
Laryngeal squamous cell carcinoma	150 patients/tissue	T-stage, Lymph node metastasis, Histological grade (all P=0.000)	Poor DFS (P=0.000)	Kaplan-Meier and multivariate Cox regression analysis	Yes	55
Laryngeal squamous cell carcinoma	188 patients/tissue	Age, primary cancer site, differentiation, T staging, cervical lymph node metastasis,	Poor outcome (P<0.001)	Kaplan-Meier and Cox regression analysis	Yes	56

		clinical stage, smoke preoperatively (all P<0.05)				
Melanomas	187 patients/tissue	Metastasis (P=0.034)	Not correlated with survival (P=0.067)	Kaplan-Meier analysis	Nd	57
Nasopharyngeal carcinoma	161 patients/tissue	Clinical stage (P<0.001) and N classification (P<0.001)	Poor OS and DFS (all P<0.001)	Kaplan-Meier and multivariate Cox regression analysis	Yes	58
Lung cancer	84 patients/tissue	Age, clinical stages, and lymph node metastases (all P<0.05)	Short survival times (P=0.004)	Kaplan-Meier and multivariate Cox regression analysis	Nd	59
Lung cancer (small-size pulmonary adenocarcinomas)	49 patients/tissue	Lymph node metastasis (P=0.0007), lymphovascular invasion (P=0.0084) and a higher stage (P=0.05)	Nd	Nd	Nd	60
Non-small cell lung cancer	81 patients/tissue	Neoplasm stage, mediastinal lymph node metastases (all, P<0.05)	Poor prognosis for median survival (P<0.05)	Kaplan-Meier analysis	Nd	61
Non-small cell lung cancer	378 patients/tissue; 154 patients/serum	Nd	Higher relapse (P=0.046)	chi-square test	Nd	62
Non-small cell lung cancer	61 patients/tissue	Tumor diameter (P<0.05) and mediastinal lymph node metastasis (P<0.05)	Nd	Nd	Nd	63
Non-small cell lung cancer	156 patients/tissue	Differentiated degree, clinical stage, N classification, and M classification (all P<0.05)	Poor OS (P<0.001)	Kaplan-Meier and Cox regression analysis	Yes	64
Non-small cell lung cancer	128 patients/tissue	Lymph node metastasis (P=0.022) and TNM stage (P=0.042)	Shorter OS (P<0.05)	Kaplan-Meier and Cox regression analysis	Yes	65
Non-small cell lung cancer	220 patients/tissue	High tumor grade (P=0.017) and proliferation (P=0.021)	Shorter OS and DFS (all P<0.05)	Kaplan-Meier and Cox regression analysis	Yes	66
Non-small-cell lung cancer	501 patients/serum	Lymphatic (P<0.001) and distant metastases (P<0.001)	Lower OS rate (P=0.044)	Kaplan-Meier and Cox regression analysis	Yes	67
Non-small-cell lung cancer	98 patients/tissue	TNM stage and regional lymph node metastasis (all P<0.05)	Nd	Nd	Nd	68
Oral and oropharyngeal squamous cell carcinomas	129 patients/tissue	Size or extent of the tumor (P<0.001), positive lymph node metastasis (P<0.001), distant	Nd	Nd	Nd	69

		metastasis (P=0.014) and clinical staging (P<0.001)				
Oral squamous cell carcinoma	113 patients/tissue	Not associated with age, tumor size, histopathological grade, clinical TNM stage, and recurrence	Poor DFS (p<0.001)	Kaplan-Meier and Cox regression analysis	Yes	70
Oral squamous cell carcinoma	40 patients/tissue	Tumor staging (p=0.01), tumor size (p=0.03), and lymph node staging (p<0.001)	Shorter OS (p=0.005)	Kaplan-Meier and Cox regression analysis	Yes	71
Oral squamous cell carcinoma	131 patients/tissue	Tumor stage (P=0.041), increased lymph node metastasis (P=0.001), less differentiation (P=0.005),<br>increased recurrence (P=0.038)	Shorter OS (P=0.004) and DFS (P=0.013)	Kaplan-Meier analysis	Nd	72
Oral squamous cell carcinoma	46 patients/tissue	Nodal metastasis (P=0.027), tumor recurrence (p<0.001)	Poor OS (P=0.013)	Kaplan-Meier and Cox regression analysis	Yes	73
Ovarian cancer (ovarian surface epithelial carcinomas)	172 patients/tissue	Histological grades (P<0.001) and clinical stages (P<0.05)	Poorer survival (P<0.001)	Kaplan-Meier analysis	Nd	74
Ovarian cancer (serous ovarian cancer)	56 patients/tissue	Ki-67 expression (p=0.016)	Reduced OS (P=0.032)	Kaplan-Meier and multivariate Cox regression analysis	Yes	75
Ovarian cancer (Borderline ovarian tumours)	140 patients/tissue	Serous subtype (P<0.001) and micropapillary pattern (P<0.001), the presence of implants (P=0.022), and higher FIGO stage (P=0.020).	Nd	Nd	Nd	76
Ovarian cancer (epithelial ovarian cancer)	89 patients/tissue	The occurrence of residual postoperative tumor >1 cm (P=0.04) and serous subtype of carcinoma (P=0.05)	Poor OS (p=0.02)	Kaplan–Meier analysis	Nd	77
Ovarian cancer (serous ovarian carcinoma)	79 patients/tissue	Lymph node involvement, distance metastasis and FIGO stage (III/IV) (all P<0.05)	Poor OS (P=0.010) and shorter PFS (P<0.001)	Kaplan-Meier and Cox regression analysis	Yes	78
Ovarian cancer (mucinous cystadenocarcinomas)	47 patients/tissue	T stage, N stage, AJCC clinical stage (all P<0.05)	Poorer survival rates (P<0.001)	Kaplan-Meier analysis	No	79
Osteosarcoma	67 patients/tissue	Nd	shorter overall survival (P<0.01)	Kaplan-Meier survival analysis	No	80

Pancreatic and ampulla of vater adenocarcinomas	90 patients/tissue	Histological grade, AJCC stage (all P<0.05)	Shorter survival rate (P=0.04)	Kaplan-Meier analysis	Nd	81
Pancreatic intraepithelial neoplasia	70 patients/tissue	Grade of PanIN (P < 0.001)	Nd	Nd	Nd	82
Pancreatobiliary adenocarcinoma	100 patients/tissue	Advanced grades, advanced T stages (all P<0.05).	Shorter survival times (P<0.05)	Kaplan-Meier analysis	Nd	83
Prostate cancer	196 patients/tissue	Increased rate of prostate-specific antigen recurrence following radical prostatectomy (P=0.075)	Lower-probability DFS (P=0.075)	Kaplan-Meier analysis	Nd	84
Renal cell carcinoma	100 patients/tissue	Histological grades and clinical stages (P<0.05)	Poorer survival (P<0.05)	Kaplan-Meier analysis	Nd	85
Renal cell carcinoma	194 patients/tissue	Not associated with age, tumor size, and clinical TNM stage.	Poor OS (P=0.004) and RFS (p=0.0005)	Kaplan-Meier analysis	Yes	86
Renal cell carcinoma	136 primary and 54 metastatic specimens	High tumor stage (P=0.008), high tumor grade (P=0.002), large tumor size (P<0.001)	Poor MFS (P<0.001)	Kaplan-Meier and multivariate Cox regression analysis	Yes	87
Skull base chordoma	34 patients/tissue	Tumor recurrence and high invasiveness (all P<0.05)	Nd	Nd	Nd	88
Small intestinal carcinomas	194 patients/tissue	Poorly and undifferentiated histology (P<0.001) and lymphatic invasion (P=0.019)	Shorter OS (P=0.001)	Kaplan-Meier analysis	Yes	89
Soft tissue sarcomas	249 patients/tissue	Histological grade (P<0.05)	Shorter DSS (P=0.006)	Kaplan-Meier and univariate Cox regression analysis	No	90
Thyroid neoplasms	138 patients/tissue	Ki-67 labeling index (P=0.0006) and lymph node metastasis (P=0.0406)	No significant difference on 3-year OS	Nd	Nd	91
Tongue squamous cell carcinoma	106 patients/tissue	N classification (P = 0.016), clinical stage (P = 0.047) and relapse (P = 0.003)	Poor OS (P=0.055) and DFS (P=0.003)	Kaplan-Meier and Cox regression analysis	Nd	92
Uterine carcinosarcoma	44 patients/tissue	Extrapelvic disease (P=0.028), Higher stage (P=0.021), larger tumor size (P=0.032)	Shorter PFS (P=0.035)	Kaplan-Meier analysis	Nd	93

Note: ER, estrogen receptor; PR, progesterone receptor; HER2, human epidermal growth factor receptor 2; FIGO, International Federation of Gynecology and Obstetrics; AJCC, American Joint Committee on Cancer; RFS, relapse/recurrence-free survival; OS, overall survival; PFS, progression-free survival; DFS, disease-free survival; DSS, disease-specifc survival; MFS, metastasis-free survival; Nd, not described.

REFERENCES

1. Poli, G., Ruggiero, C., Cantini, G., Canu, L., Baroni, G., Armignacco, R., Jouinot, A., Santi, R., Ercolino, T., Ragazzon, B., et al. (2019). Fascin-1 Is a Novel Prognostic Biomarker Associated With Tumor Invasiveness in Adrenocortical Carcinoma. J. Clin. Endocrinol. Metab.. *104*, 1712-1724.
2. Liang, J., Liu, Z., Wei, X., Zhou, L., Tang, Y., Zhou, C., Wu, K., Zhang, F., Zhang, F., Lu, Y., et al. (2019). Expression of FSCN1 and FOXM1 are associated with poor prognosis of adrenocortical carcinoma patients. BMC Cancer. *19*, 1165.
3. Bi, J., Chen, X., Zhang, Y., Li, B., Sun, J., Shen, H., and Kong, C. (2012). Fascin is a predictor for invasiveness and recurrence of urothelial carcinoma of bladder. Urol. Oncol.. *30*, 688-694.
4. Gomaa, W., Al-Maghrabi, H., Al-Attas, M., Al-Ghamdi, F., and Al-Maghrabi, J. (2019). Fascin expression in urinary bladder urothelial carcinoma correlates with unfavourable prognosis. Int J Clin Exp Pathol. *12*, 3901-3907.
5. El-Rehim, D.M., El-Maqsoud, N.M., El-Hamid, A.M., El-Bab, T.K., and Galal, E.M. (2013). Expression of extracellular matrix metalloproteinase inducer and fascin in urinary bladder cancer: Correlation with clinicopathological characteristics. Mol Clin Oncol. *1*, 297-304.
6. Bi, J.B., Zhu, Y., Chen, X.L., Yu, M., Zhang, Y.X., Li, B.X., Sun, J.W., Shen, H.L., and Kong, C.Z. (2013). The role of fascin in migration and invasion of urothelial carcinoma of the bladder. Urol. Int.. *91*, 227-235.
7. Tampaki, E.C., Tampakis, A., Nonni, A., von Flüe, M., Patsouris, E., Kontzoglou, K., and Kouraklis, G. (2019). Combined Fascin-1 and MAP17 Expression in Breast Cancer Identifies Patients with High Risk for Disease Recurrence. Mol Diagn Ther. *23*, 635-644.
8. Abbasi, A., Noroozinia, F., Anvar, S., Abbasi, M., Hosseinzadeh, S., and Mokhtari, S. (2019). Fascin overexpression is associated with higher grades of breast cancer. Polish journal of pathology : official journal of the Polish Society of Pathologists. *70*, 264-268.
9. Esnakula, A.K., Ricks-Santi, L., Kwagyan, J., Kanaan, Y.M., DeWitty, R.L., Wilson, L.L., Gold, B., Frederick, W.A., and Naab, T.J. (2014). Strong association of fascin expression with triple negative breast cancer and basal-like phenotype in African-American women. J. Clin. Pathol.. *67*, 153-160.
10. Lee, H.J., An, H.J., Kim, T.H., Kim, G., Kang, H., Heo, J.H., Kwon, A.Y., and Kim, S. (2017). Fascin expression is inversely correlated with breast cancer metastasis suppressor 1 and predicts a worse survival outcome in node-negative breast cancer patients. J Cancer. *8*, 3122-3129.
11. Ghebeh, H., Al-Khaldi, S., Olabi, S., Al-Dhfyan, A., Al-Mohanna, F., Barnawi, R., Tulbah, A., Al-Tweigeri, T., Ajarim, D., and Al-Alwan, M. (2014). Fascin is involved in the chemotherapeutic resistance of breast cancer cells predominantly via the PI3K/Akt pathway. Br. J. Cancer. *111*, 1552-1561.
12. Min, K.W., Kim, D.H., Do, S.I., Chae, S.W., Kim, K., Sohn, J.H., Pyo, J.S., Lee, H.J., Kim, D.H., Oh, S., et al. (2016). Negative association between GATA3 and fascin could predict relapse-free and overall survival in patients with breast cancer. Virchows Arch.. *468*, 409-416.
13. Min, K.W., Chae, S.W., Kim, D.H., DO, S.I., Kim, K., Lee, H.J., Sohn, J.H., Pyo, J.S., Kim, D.H., Oh, S., et al. (2015). Fascin expression predicts an aggressive clinical course in patients with advanced breast cancer. Oncol Lett. *10*, 121-130.
14. Wang, C.Q., Li, Y., Huang, B.F., Zhao, Y.M., Yuan, H., Guo, D., Su, C.M., Hu, G.N., Wang, Q., Long, T., et al. (2017). EGFR conjunct FSCN1 as a Novel Therapeutic Strategy in Triple-Negative Breast Cancer. Sci Rep. *7*, 15654.
15. Omran, O.M., and Al Sheeha, M. (2015). Cytoskeletal Focal Adhesion Proteins Fascin-1 and Paxillin Are Predictors of Malignant Progression and Poor Prognosis in Human Breast Cancer. J. Environ. Pathol. Toxicol. Oncol.. *34*, 201-212.
16. Youssef, N.S., and Hakim, S.A. (2014). Association of Fascin and matrix metalloproteinase-9 expression with poor prognostic parameters in breast carcinoma of Egyptian women. Diagn Pathol. *9*, 136.
17. Yoder, B.J., Tso, E., Skacel, M., Pettay, J., Tarr, S., Budd, T., Tubbs, R.R., Adams, J.C., and Hicks, D.G. (2005). The expression of fascin, an actin-bundling motility protein, correlates

with hormone receptor-negative breast cancer and a more aggressive clinical course. Clin. Cancer Res.. *11*, 186-192.

18. Al-Alwan, M., Olabi, S., Ghebeh, H., Barhoush, E., Tulbah, A., Al-Tweigeri, T., Ajarim, D., and Adra, C. (2011). Fascin is a key regulator of breast cancer invasion that acts via the modification of metastasis-associated molecules. PLoS ONE. *6*, e27339.
19. Mao, X., Chen, D., Wu, J., Li, J., Zhou, H., Wu, Y., and Duan, X. (2013). Differential expression of fascin, E-cadherin and vimentin: Proteins associated with survival of cholangiocarcinoma patients. Am. J. Med. Sci.. *346*, 261-268.
20. Iguchi, T., Aishima, S., Taketomi, A., Nishihara, Y., Fujita, N., Sanefuji, K., Sugimachi, K., Yamashita, Y., Maehara, Y., and Tsuneyoshi, M. (2009). Fascin overexpression is involved in carcinogenesis and prognosis of human intrahepatic cholangiocarcinoma: immunohistochemical and molecular analysis. Hum. Pathol.. *40*, 174-180.
21. Binnetoglu, A., Sari, M., Baglam, T., Erbarut Seven, I., Yumusakhuylu, A.C., Topuz, M.F., and Batman, C. (2015). Fascin expression in cholesteatoma: correlation with destruction of the ossicular chain and extent of disease. Clin Otolaryngol. *40*, 335-340.
22. Puppa, G., Maisonneuve, P., Sonzogni, A., Masullo, M., Chiappa, A., Valerio, M., Zampino, M.G., Franceschetti, I., Capelli, P., Chilosi, M., et al. (2007). Independent prognostic value of fascin immunoreactivity in stage III-IV colonic adenocarcinoma. Br. J. Cancer. *96*, 1118-1126.
23. Hashimoto, Y., Skacel, M., Lavery, I.C., Mukherjee, A.L., Casey, G., and Adams, J.C. (2006). Prognostic significance of fascin expression in advanced colorectal cancer: an immunohistochemical study of colorectal adenomas and adenocarcinomas. BMC Cancer. *6*, 241.
24. Oh, S.Y., Kim, Y.B., Suh, K.W., Paek, O.J., and Moon, H.Y. (2012). Prognostic impact of fascin-1 expression is more significant in advanced colorectal cancer. J. Surg. Res.. *172*, 102-108.
25. Piskor, B.M., Pryczynicz, A., Lubowicka, E., Miniewska, K., Zinczuk, J., Zareba, K., and Guzinska-Ustymowicz, K. (2018). Immunohistochemical expression of Fascin-1 in colorectal cancer in relation to clinical and pathological parameters. Folia Histochem. Cytobiol.. *1*, 106-112.
26. Alajez, N.M. (2016). Significance of BMI1 and FSCN1 expression in colorectal cancer. Saudi J Gastroenterol. *22*, 288-293.
27. Chan, C., Jankova, L., Fung, C.L., Clarke, C., Robertson, G., Chapuis, P.H., Bokey, L., Lin, B.P., Dent, O.F., and Clarke, S. (2010). Fascin expression predicts survival after potentially curative resection of node-positive colon cancer. Am. J. Surg. Pathol.. *34*, 656-666.
28. Ozerhan, I.H., Ersoz, N., Onguru, O., Ozturk, M., Kurt, B., and Cetiner, S. (2010). Fascin expression in colorectal carcinomas. Clinics (Sao Paulo). *65*, 157-164.
29. Ou, C., Sun, Z., He, X., Li, X., Fan, S., Zheng, X., Peng, Q., Li, G., Li, X., and Ma, J. (2020). Targeting YAP1/LINC00152/FSCN1 Signaling Axis Prevents the Progression of Colorectal Cancer. Adv Sci (Weinh). *7*, 1901380.
30. Jung, E.J., Lee, J.H., Min, B.W., Kim, Y.S., and Choi, J.S. (2011). Clinicopathologic significance of fascin, extracellular matrix metalloproteinase inducer, and ezrin expressions in colorectal adenocarcinoma. Indian J Pathol Microbiol. *54*, 32-36.
31. Gun, B.D., Bahadir, B., Bektas, S., Barut, F., Yurdakan, G., Kandemir, N.O., and Ozdamar, S.O. (2012). Clinicopathological significance of fascin and CD44v6 expression in endometrioid carcinoma. Diagn Pathol. *7*, 80.
32. Chen, W.X., Hong, X.B., Hong, C.Q., Liu, M., Li, L., Huang, L.S., Xu, L.Y., Xu, Y.W., Peng, Y.H., and Li, E.M. (2017). Tumor-associated autoantibodies against Fascin as a novel diagnostic biomarker for esophageal squamous cell carcinoma. Clin Res Hepatol Gastroenterol. *41*, 327-332.
33. Lin, C., Zhang, S., Wang, Y., Wang, Y., Nice, E., Guo, C., Zhang, E., Yu, L., Li, M., Liu, C., et al. (2018). Functional Role of a Novel Long Noncoding RNA TTN-AS1 in Esophageal Squamous Cell Carcinoma Progression and Metastasis. Clin. Cancer Res.. *24*, 486-498.
34. Takikita, M., Hu, N., Shou, J.Z., Giffen, C., Wang, Q.H., Wang, C., Hewitt, S.M., and Taylor, P.R. (2011). Fascin and CK4 as biomarkers for esophageal squamous cell carcinoma. Anticancer Res.. *31*, 945-952.
35. Zhao, Q., Shen, J.H., Shen, Z.Y., Wu, Z.Y., Xu, X.E., Xie, J.J., Wu, J.Y., Huang, Q., Lu, X.F., Li, E.M., et al. (2010). Phosphorylation of fascin decreases the risk of poor survival in patients with esophageal squamous cell carcinoma. J. Histochem. Cytochem.. *58*, 979-988.

36. Hashimoto, Y., Ito, T., Inoue, H., Okumura, T., Tanaka, E., Tsunoda, S., Higashiyama, M., Watanabe, G., Imamura, M., and Shimada, Y. (2005). Prognostic significance of fascin overexpression in human esophageal squamous cell carcinoma. Clin. Cancer Res.. *11*, 2597-2605.
37. Won, K.Y., Kim, G.Y., Lim, S.J., Park, Y.K., and Kim, Y.W. (2009). Prognostic significance of fascin expression in extrahepatic bile duct carcinomas. Pathol. Res. Pract.. *205*, 742-748.
38. Roh, Y.H., Kim, Y.H., Choi, H.J., Lee, K.E., and Roh, M.S. (2009). Fascin overexpression correlates with positive thrombospondin-1 and syndecan-1 expressions and a more aggressive clinical course in patients with gallbladder cancer. J Hepatobiliary Pancreat Surg. *16*, 315-321.
39. Tsai, W.C., Jin, J.S., Chang, W.K., Chan, D.C., Yeh, M.K., Cherng, S.C., Lin, L.F., Sheu, L.F., and Chao, Y.C. (2007). Association of cortactin and fascin-1 expression in gastric adenocarcinoma: correlation with clinicopathological parameters. J. Histochem. Cytochem.. *55*, 955-962.
40. Son, B.K., Kim, D.H., Min, K.W., Kim, E.K., and Kwon, M.J. (2018). Smad4/Fascin index is highly prognostic in patients with diffuse type EBV-associated gastric cancer. Pathol. Res. Pract.. *214*, 475-481.
41. Zheng, H.C., and Zhao, S. (2017). The meta and bioinformatics analysis of fascin expression in gastric cancer: a potential marker for aggressiveness and worse prognosis. Oncotarget. *8*, 105574-105583.
42. Tu, L., Xu, J., Wang, M., Zhao, W.Y., Zhang, Z.Z., Zhu, C.C., Tang, D.F., Zhang, Y.Q., Wang, D.H., Zuo, J., et al. (2016). Correlations of fascin-1 and cadherin-17 protein expression with clinicopathologic features and prognosis of patients with gastric cancer. Tumour Biol.. *37*, 8775-8782.
43. Kim, S.J., Kim, D.C., Kim, M.C., Jung, G.J., Kim, K.H., Jang, J.S., Kwon, H.C., Kim, Y.M., and Jeong, J.S. (2012). Fascin expression is related to poor survival in gastric cancer. Pathol. Int.. *62*, 777-784.
44. Hashimoto, Y., Shimada, Y., Kawamura, J., Yamasaki, S., and Imamura, M. (2004). The prognostic relevance of fascin expression in human gastric carcinoma. Oncology. *67*, 262-270.
45. Yamamoto, H., Kohashi, K., Fujita, A., and Oda, Y. (2013). Fascin-1 overexpression and miR-133b downregulation in the progression of gastrointestinal stromal tumor. Mod. Pathol.. *26*, 563-571.
46. Park, K.S., Lee, H.W., Park, S.H., Park, T.I., and Hwang, J.H. (2016). The clinical significance of fascin expression in a newly diagnosed primary glioblastoma. J. Neurooncol.. *129*, 495-503.
47. Zhang, H., Cong, Q.X., Zhang, S.G., Zhai, X.W., Li, H.F., and Li, S.Q. (2018). High Expression Levels of Fascin-1 Protein in Human Gliomas and its Clinical Relevance. Open Med (Wars). *13*, 544-550.
48. Gunal, A., Onguru, O., Safali, M., and Beyzadeoglu, M. (2008). Fascin expression [corrected] in glial tumors and its prognostic significance in glioblastomas. Neuropathology. *28*, 382-386.
49. Lee, L.Y., Chen, Y.J., Lu, Y.C., Liao, C.T., Chen, I.H., Chang, J.T., Huang, Y.C., Chen, W.H., Huang, C.C., Tsai, C.Y., et al. (2015). Fascin is a circulating tumor marker for head and neck cancer as determined by a proteomic analysis of interstitial fluid from the tumor microenvironment. Clin. Chem. Lab. Med.. *53*, 1631-1641.
50. Papaspyrou, K., Brochhausen, C., Schmidtmann, I., Fruth, K., Gouveris, H., Kirckpatrick, J., Mann, W., and Brieger, J. (2014). Fascin upregulation in primary head and neck squamous cell carcinoma is associated with lymphatic metastasis. Oncol Lett. *7*, 2041-2046.
51. Huang, X., Ji, J., Xue, H., Zhang, F., Han, X., Cai, Y., Zhang, J., and Ji, G. (2012). Fascin and cortactin expression is correlated with a poor prognosis in hepatocellular carcinoma. Eur J Gastroenterol Hepatol. *24*, 633-639.
52. Iguchi, T., Aishima, S., Umeda, K., Sanefuji, K., Fujita, N., Sugimachi, K., Gion, T., Taketomi, A., Maehara, Y., and Tsuneyoshi, M. (2009). Fascin expression in progression and prognosis of hepatocellular carcinoma. J Surg Oncol. *100*, 575-579.
53. Gao, W., Zhang, C., Feng, Y., Chen, G., Wen, S., Huangfu, H., and Wang, B. (2012). Fascin-1, ezrin and paxillin contribute to the malignant progression and are predictors of clinical prognosis in laryngeal squamous cell carcinoma. PLoS ONE. *7*, e50710.

54. Durmaz, A., Kurt, B., Ongoru, O., Karahatay, S., Gerek, M., and Yalcin, S. (2010). Significance of fascin expression in laryngeal squamous cell carcinoma. J Laryngol Otol. *124*, 194-198.
55. Zou, J., Yang, H., Chen, F., Zhao, H., Lin, P., Zhang, J., Ye, H., Wang, L., and Liu, S. (2010). Prognostic significance of fascin-1 and E-cadherin expression in laryngeal squamous cell carcinoma. Eur. J. Cancer Prev.. *19*, 11-17.
56. Gao, W., Zhang, C., Li, W., Li, H., Sang, J., Zhao, Q., Bo, Y., Luo, H., Zheng, X., Lu, Y., et al. (2019). Promoter Methylation-Regulated miR-145-5p Inhibits Laryngeal Squamous Cell Carcinoma Progression by Targeting FSCN1. Mol. Ther.. *27*, 365-379.
57. Ma, Y., Faller, W.J., Sansom, O.J., Brown, E.R., Doig, T.N., Melton, D.W., and Machesky, L.M. (2015). Fascin expression is increased in metastatic lesions but does not correlate with progression nor outcome in melanoma. Melanoma Res.. *25*, 169-172.
58. Wu, D., Chen, L., Liao, W., Ding, Y., Zhang, Q., Li, Z., and Liu, L. (2010). Fascin1 expression predicts poor prognosis in patients with nasopharyngeal carcinoma and correlates with tumor invasion. Ann. Oncol.. *21*, 589-596.
59. Zhao, W., Gao, J., Wu, J., Liu, Q.H., Wang, Z.G., Li, H.L., and Xing, L.H. (2015). Expression of Fascin-1 on human lung cancer and paracarcinoma tissue and its relation to clinicopathological characteristics in patients with lung cancer. Onco Targets Ther. *8*, 2571-2576.
60. Choi, P.J., Yang, D.K., Son, C.H., Lee, K.E., Lee, J.I., and Roh, M.S. (2006). Fascin immunoreactivity for preoperatively predicting lymph node metastases in peripheral adenocarcinoma of the lung 3 cm or less in diameter. Eur J Cardiothorac Surg. *30*, 538-542.
61. Zhang, Y., Liang, B., and Dong, H. (2018). Expression of fascin_1 protein in cancer tissues of patients with nonsmall cell lung cancer and its relevance to patients' clinicopathologic features and prognosis. J Cancer Res Ther. *14*, 856-859.
62. Yang, L., Teng, Y., Han, T.P., Li, F.G., Yue, W.T., and Wang, Z.T. (2017). Clinical significance of fascin-1 and laminin-5 in non-small cell lung cancer. Genet. Mol. Res.. *16*.
63. Zhang, J., Wang, X., Zhang, Y., Wu, J., and Zhou, N. (2016). Leucine-rich repeats and immunoglobulin-like domains protein 1 and fascin actin-bundling protein 1 expression in nonsmall cell lung cancer. J Cancer Res Ther. *12*, C248-248C251.
64. Luo, A., Yin, Y., Li, X., Xu, H., Mei, Q., and Feng, D. (2015). The clinical significance of FSCN1 in non-small cell lung cancer. Biomed. Pharmacother.. *73*, 75-79.
65. Ling, X.L., Zhang, T., Hou, X.M., and Zhao, D. (2015). Clinicopathological significance of fascin-1 expression in patients with non-small cell lung cancer. Onco Targets Ther. *8*, 1589-1595.
66. Pelosi, G., Pastorino, U., Pasini, F., Maissoneuve, P., Fraggetta, F., Iannucci, A., Sonzogni, A., De Manzoni, G., Terzi, A., Durante, E., et al. (2003). Independent prognostic value of fascin immunoreactivity in stage I nonsmall cell lung cancer. Br. J. Cancer. *88*, 537-547.
67. Teng, Y., Xu, S., Yue, W., Ma, L., Zhang, L., Zhao, X., Guo, Y., Zhang, C., Gu, M., and Wang, Y. (2013). Serological investigation of the clinical significance of fascin in non-small-cell lung cancer. Lung Cancer. *82*, 346-352.
68. Zhao, J., Zhou, Y., Zhang, Z., Tian, F., Ma, N., Liu, T., Gu, Z., and Wang, Y. (2010). Upregulated fascin1 in non-small cell lung cancer promotes the migration and invasiveness, but not proliferation. Cancer Lett.. *290*, 238-247.
69. Chen, S.F., Yang, S.F., Li, J.W., Nieh, P.C., Lin, S.Y., Fu, E., Bai, C.Y., Jin, J.S., Lin, C.Y., and Nieh, S. (2007). Expression of fascin in oral and oropharyngeal squamous cell carcinomas has prognostic significance - a tissue microarray study of 129 cases. Histopathology. *51*, 173-183.
70. Rodrigues, P.C., Sawazaki-Calone, I., Ervolino de Oliveira, C., Soares Macedo, C.C., Dourado, M.R., Cervigne, N.K., Miguel, M.C., Ferreira do Carmo, A., Lambert, D.W., Graner, E., et al. (2017). Fascin promotes migration and invasion and is a prognostic marker for oral squamous cell carcinoma. Oncotarget. *8*, 74736-74754.
71. Routray, S., Kheur, S., Chougule, H.M., Mohanty, N., and Dash, R. (2017). Establishing Fascin over-expression as a strategic regulator of neoplastic aggression and lymph node metastasis in oral squamous cell carcinoma tumor microenvironment. Ann Diagn Pathol. *30*, 36-41.
72. Alam, H., Bhate, A.V., Gangadaran, P., Sawant, S.S., Salot, S., Sehgal, L., Dange, P.P., Chaukar, D.A., D'cruz, A.K., Kannanl, S., et al. (2012). Fascin overexpression promotes

neoplastic progression in oral squamous cell carcinoma. BMC Cancer. *12*, 32.
73. Lee, T.K., Poon, R.T., Man, K., Guan, X.Y., Ma, S., Liu, X.B., Myers, J.N., and Yuen, A.P. (2007). Fascin over-expression is associated with aggressiveness of oral squamous cell carcinoma. Cancer Lett.. *254*, 308-315.
74. Lin, C.K., Su, H.Y., Tsai, W.C., Sheu, L.F., and Jin, J.S. (2008). Association of cortactin, fascin-1 and epidermal growth factor receptor (EGFR) expression in ovarian carcinomas: correlation with clinicopathological parameters. Dis. Markers. *25*, 17-26.
75. Daponte, A., Kostopoulou, E., Papandreou, C.N., Daliani, D.D., Minas, M., Koukoulis, G., and Messinis, I.E. (2008). Prognostic significance of fascin expression in advanced poorly differentiated serous ovarian cancer. Anticancer Res.. *28*, 1905-1910.
76. El-Balat, A., Arsenic, R., Sänger, N., Karn, T., Becker, S., Holtrich, U., and Engels, K. (2016). Fascin-1 expression as stratification marker in borderline epithelial tumours of the ovary. J. Clin. Pathol.. *69*, 142-148.
77. Hanker, L.C., Karn, T., Holtrich, U., Graeser, M., Becker, S., Reinhard, J., Ruckhäberle, E., Gevensleben, H., and Rody, A. (2013). Prognostic impact of fascin-1 (FSCN1) in epithelial ovarian cancer. Anticancer Res.. *33*, 371-377.
78. Park, S.H., Song, J.Y., Kim, Y.K., Heo, J.H., Kang, H., Kim, G., An, H.J., and Kim, T.H. (2014). Fascin1 expression in high-grade serous ovarian carcinoma is a prognostic marker and knockdown of fascin1 suppresses the proliferation of ovarian cancer cells. Int. J. Oncol.. *44*, 637-646.
79. Lin, C.K., Chao, T.K., Yu, C.P., Yu, M.H., and Jin, J.S. (2009). The expression of six biomarkers in the four most common ovarian cancers: correlation with clinicopathological parameters. APMIS. *117*, 162-175.
80. Arlt, M.J., Kuzmanov, A., Snedeker, J.G., Fuchs, B., Silvan, U., and Sabile, A.A. (2019). Fascin-1 enhances experimental osteosarcoma tumor formation and metastasis and is related to poor patient outcome. BMC Cancer. *19*, 83.
81. Tsai, W.C., Lin, C.K., Lee, H.S., Gao, H.W., Nieh, S., Chan, D.C., and Jin, J.S. (2013). The correlation of cortactin and fascin-1 expression with clinicopathological parameters in pancreatic and ampulla of Vater adenocarcinoma. APMIS. *121*, 171-181.
82. Misiura, M., Zińczuk, J., Zaręba, K., Kamińska, D., Guzińska-Ustymowicz, K., and Pryczynicz, A. (2020). Actin-Bundling Proteins (Actinin-4 and Fascin-1) are Involved in the Development of Pancreatic Intraepithelial Neoplasia (PanIN). Am. J. Med. Sci.. *359*, 147-155.
83. Tsai, W.C., Chao, Y.C., Sheu, L.F., Lin, Y.F., Nieh, S., Chen, A., Yu, C.P., and Jin, J.S. (2007). EMMPRIN and fascin overexpression associated with clinicopathologic parameters of pancreatobiliary adenocarcinoma in Chinese people. APMIS. *115*, 929-938.
84. Darnel, A.D., Behmoaram, E., Vollmer, R.T., Corcos, J., Bijian, K., Sircar, K., Su, J., Jiao, J., Alaoui-Jamali, M.A., and Bismar, T.A. (2009). Fascin regulates prostate cancer cell invasion and is associated with metastasis and biochemical failure in prostate cancer. Clin. Cancer Res.. *15*, 1376-1383.
85. Tsai, W.C., Sheu, L.F., Nieh, S., Yu, C.P., Sun, G.H., Lin, Y.F., Chen, A., and Jin, J.S. (2007). Association of EMMPRIN and fascin expression in renal cell carcinoma: correlation with clinicopathological parameters. World J Urol. *25*, 73-80.
86. Zhang, M., Zhao, Z., Duan, X., Chen, P., Peng, Z., and Qiu, H. (2018). FSCN1 predicts survival and is regulated by a PI3K-dependent mechanism in renal cell carcinoma. J. Cell. Physiol.. *233*, 4748-4758.
87. Zigeuner, R., Droschl, N., Tauber, V., Rehak, P., and Langner, C. (2006). Biologic significance of fascin expression in clear cell renal cell carcinoma: systematic analysis of primary and metastatic tumor tissues using a tissue microarray technique. Urology. *68*, 518-522.
88. Gao, Z., Zhang, Q., Kong, F., Chen, G., Li, M., Guo, H., Liang, J., Bao, Y., and Ling, F. (2012). Fascin expression in skull base chordoma: correlation with tumor recurrence and dura erosion. Med. Oncol.. *29*, 2438-2444.
89. Gu, M.J., Kim, J.Y., and Park, J.B. (2014). Fascin expression predicts lymph node metastasis and worse survival in small intestinal carcinoma. Pathology. *46*, 21-24.
90. Valkov, A., Sorbye, S.W., Kilvaer, T.K., Donnem, T., Smeland, E., Bremnes, R.M., and Busund, L.T. (2011). The prognostic impact of TGF-β1, fascin, NF-κB and PKC-ζ expression

in soft tissue sarcomas. PLoS ONE. *6*, e17507.

91. Chen, G., Zhang, F.R., Ren, J., Tao, L.H., Shen, Z.Y., Lv, Z., Yu, S.J., Dong, B.F., Xu, L.Y., and Li, E.M. (2008). Expression of fascin in thyroid neoplasms: a novel diagnostic marker. J. Cancer Res. Clin. Oncol.. *134*, 947-951.
92. Chen, Y., Tian, T., Li, Z.Y., Wang, C.Y., Deng, R., Deng, W.Y., Yang, A.K., Chen, Y.F., and Li, H. (2019). FSCN1 is an effective marker of poor prognosis and a potential therapeutic target in human tongue squamous cell carcinoma. Cell Death Dis. *10*, 356.
93. Richmond, A.M., Blake, E.A., Torkko, K., Smith, E.E., Spillman, M.A., and Post, M.D. (2017). Fascin Is Associated With Aggressive Behavior and Poor Outcome in Uterine Carcinosarcoma. Int. J. Gynecol. Cancer. *27*, 1895-1903.
